# Comprehensive Atlas of Wheat (*Triticum aestivum L*.) *AUXIN RESPONSE FACTOR* Expression During Male Reproductive Development and Abiotic Stress

**DOI:** 10.3389/fpls.2020.586144

**Published:** 2020-09-30

**Authors:** Lei Xu, Dezhou Wang, Shan Liu, Zhaofeng Fang, Shichao Su, Chunman Guo, Changping Zhao, Yimiao Tang

**Affiliations:** ^1^The Municipal Key Laboratory of the Molecular Genetics of Hybrid Wheat, Beijing Engineering Research Center for Hybrid, Beijing Academy of Agriculture and Forestry Sciences, Beijing, China; ^2^Peking-Tsinghua Center for Life Sciences, Academy for Advanced Interdisciplinary Studies, Peking University, Beijing, China

**Keywords:** auxin response factor, male reproductive development, abiotic stress, abscisic acid, thermosensitive genic male sterile (TGMS)

## Abstract

AUXIN RESPONSE FACTOR (ARF) proteins regulate a wide range of signaling pathways, from general plant growth to abiotic stress responses. Here, we performed a genome-wide survey in wheat (*Triticum aestivum*) and identified 69 *TaARF* members that formed 24 homoeologous groups. Phylogenetic analysis clustered *TaARF* genes into three clades, similar to *ARF* genes in *Arabidopsis* (*Arabidopsis thaliana*) and rice (*Oryza sativa*). Structural characterization suggested that *ARF* gene structure and domain composition are well conserved between plant species. Expression profiling revealed diverse patterns of *TaARF* transcript levels across a range of developmental stages, tissues, and abiotic stresses. A number of *TaARF* genes shared similar expression patterns and were preferentially expressed in anthers. Moreover, our systematic analysis identiﬁed three anther-specific *TaARF* genes (*TaARF8*, *TaARF9*, and *TaARF21*) whose expression was signiﬁcantly altered by low temperature in thermosensitive genic male-sterile (TGMS) wheat; these *TaARF* genes are candidates to participate in the cold-induced male sterility pathway, and offer potential applications in TGMS wheat breeding and hybrid seed production. Moreover, we identified putative functions for a set of *TaARFs* involved in responses to abscisic acid and abiotic stress. Overall, this study characterized the wheat *ARF* gene family and generated several hypotheses for future investigation of ARF function during anther development and abiotic stress.

## Introduction

Auxin response factors (ARFs) regulate gene expression through binding to the auxin response element (AuxRE, sequence TGTCTC) in the promoters of auxin-responsive genes. ARF function is modulated by the Aux/IAA repressors, which are targeted for degradation upon auxin signal perception (Ulmasov et al., 1999b). Typical ARF proteins contain three conserved domains: an N-terminal DNA-binding domain (DBD), a middle region (MR), and a C-terminal interaction domain (CTD) (Tiwari et al., 2003). The DBD consists of a B3 domain and an ARF domain, while the CTD mediates homodimerization or heterodimerization; the MR confers activator or repressor activity depending on its amino acid composition (Weijers and Wagner, 2016). ARF activation domains possess a QSL-rich [glycine (Q), leucine (L), serine (S)] MR, whereas ARF repression domains are enriched in serine and in some cases proline, leucine, and/or glycine (Ulmasov et al., 1999a; Tiwari et al., 2003; Wang et al., 2005; Wilmoth et al., 2005).

ARFs play a critical role in growth responses throughout plant development, such as the control of leaf vascular patterning, apical dominance, establishment of embryonic axis, root geotropism, stem phototropism, fruit development, and the circadian control of flower opening and closure (Guilfoyle and Hagen, 2007; Chandler, 2016; Li et al., 2016). For instance, *Arabidopsis* (*Arabidopsis thaliana*) *ARF5* is specifically expressed in developing embryos and vascular tissues, and is involved in embryo patterning and vascular formation (Krogan and Berleth, 2012; Krogan et al., 2014). *Arabidopsis ARF8* is expressed in seedlings and developing ﬂowers and fruits, and in addition to being a positive regulator of adventitious rooting, a specific *ARF8* splice variant regulates stamen elongation and endothecium lignification (Nagpal et al., 2005; Gutierrez et al., 2009; Ghelli et al., 2018). Therefore, *ARF8* regulates growth in both vegetative and reproductive tissues (Nagpal et al., 2005). *Arabidopsis* ARF17 plays an essential role in primexine formation and pollen development (Yang et al., 2013; Shi et al., 2015).

In rice (*Oryza sativa*), ARFs have divergent functions in plant growth and development. For example, rice ARF1 is critical for growth of vegetative organs and seed development (Attia et al., 2009), ARF12 participates in the regulation of root elongation (Qi et al., 2012), and ARF16 regulates the phosphate starvation response (Shen et al., 2013). ARF19 has been linked to regulation of leaf angle through the control of adaxial cell division (Zhang et al., 2015).

Genome-wide identification and expression analyses of the *ARF* gene family suggest that the transcript levels of many *ARF* genes are altered in various species in response to abiotic stresses, such as drought, salt, or cold. For instance, the expression of 33 out of 51 soybean (*Glycine max*) *ARF* genes is dehydration responsive in shoots or roots (Ha et al., 2013), and *Arabidopsis ARF* genes are coordinately up-regulated during cold acclimation (Hannah et al., 2005). Many *ARF* genes are repressed or induced following cold, salt, and osmotic stress in banana (*Musa acuminata* L.) (Hu et al., 2015). Together, these data indicate that *ARF* gene family members play important roles in regulating plant growth and in responses to multiple signal transduction pathways.

Wheat (*Triticum aestivum*) is an important food crop, providing approximately 20% of our caloric intake and 25% of protein intake worldwide (www.fao.org/faostat). With the influence of increasing population and climate change, it has become even more urgent to breed novel wheat varieties that exhibit improved yield and enhanced tolerance to abiotic stresses. Moreover, the availability of additional thermosensitive genic male-sterile (TGMS) mutants in wheat will greatly help in generating hybrid wheat varieties and in functional characterization of male sterility genes. Indeed, a previous study reported that an *ARF* gene is an important component of TGMS in wheat, suggesting that characterization of other *ARF* genes may inform approaches to produce TGMS wheat (Tang et al., 2012). In addition, 23 *TaARF* members encoded by a total of 68 homoeoalleles were found to be induced in response to exogenous auxin treatment (Qiao et al., 2018). However, the functions of most *TaARF* family members in male sterility and abiotic stress remain unknown.

In this study, we identified 24 wheat *ARF* genes derived from 69 homoeoalleles, including a novel *TaARF* member, *TaARF23-A.2*. We investigated the phylogenetic relationships and evolution of *ARF* genes among wheat and its relatives, including red wild einkorn wheat (*Triticum urartu*), the wheat D genome progenitor Tausch’s goatgrass (*Aegilops tauschii*), emmer wheat (*Triticum turgidum*), and barley (*Hordeus vulgare*), identifying 19 *ARF* genes in *T. urartu*, 23 in *A. tauschii*, 43 in *T. turgidum*, and 22 in *H. vulgare*. We also determined the expression patterns of *TaARF* genes during male reproductive development and various abiotic stresses. Given the important roles of microRNAs (miRNAs), such as miR160 and miR167, and *trans*-acting small interfering RNAs (tasiRNAs), which participate in abiotic stress and anther development, we also predicted which *TaARF* genes might be targeted by miRNAs. In addition, we determined the expression profiles of *TaARF* genes in a TGMS line by RT-qPCR. We expect that the results of this study will provide critical resources for enhancing plant abiotic stress tolerance and will form a foundation for further functional analysis of the possible roles of *TaARF* genes in the molecular mechanisms of cold-induced TGMS in wheat.

## Materials and Methods

### Identification of *Auxin Response Factor* Genes From *T. aestivum*, *T. urartu*, *A. tauschii*, *T. turgidum*, and *H. vulgare*

We identified all *ARF* genes in several complete genomes with the Basic Local Alignment Tools for Proteins (BLASTP), using all publicly known ARF protein sequences from *Arabidopsis* and rice as queries against the *T. aestivum* (IWGSC), *T. urartu* (ASM34745v1), *A. tauschii* (Aet_v4.0), *T. turgidum* (Svevo.v1), and *H. vulgare* (IBSC_v2) genomes through the Ensembl Plants database (http://www.plants.ensembl.org/). All ARF proteins identified after this initial search were examined by manual curation for the functional protein domains using HMMER 3.0 (hidden Markov model, HMM) software and the NCBI conserved domain database (http://www.ncbi.nlm.nih.gov/cdd). The HMM profiles of ARF proteins (PF02309, PF02362, and PF06507; PF02309 is considered an optional domain) were extracted from the Pfam database (http://pfam.sanger.ac.uk/). The subcellular localization of TaARF proteins was predicted with CELLO (http://cello.life.nctu.edu.tw/). ARF protein molecular weights and isoelectric points were calculated with the Compute pI/Mw tool (http://web.expasy.org/compute_pi/).

### Phylogenetic and Sequence Analysis of *TaARF* Genes

We performed a multiple sequence alignment of ARF proteins from seven species (*A. thaliana*, *O. sativa*, *T. aestivum*, *T. urartu*, *A. tauschii*, *T. turgidum*, and *H. vulgare*) using MUSCLE (http://www.ebi.ac.uk/Tools/msa/muscle/). The phylogenetic tree was constructed with the MEGA 7 software using the maximum-likelihood method with JTT+G model and 1000-replicates bootstrap. Another phylogenetic tree was constructed using only wheat ARF protein sequences and aligned using MEGA 7 based on the neighbor-joining method with the following parameters: Poisson model, pairwise deletion, and 1000-replicates bootstrap. We measured the genetic variation and selective pressures of *ARF* genes among and within *T. aestivum*, *T. urartu*, *A. tauschii*, *T. turgidum*, and *H. vulgare* with the software DnaSP 5.

We retrieved the coding sequences (CDS) and genomic sequences for *TaARF* genes from the Ensembl plants database and displayed their genomic organization with the Gene Structure Display Server program (http://gsds.cbi.pku.edu.cn/). We analyzed protein motifs in *Ta*ARF proteins using MEME software (http://meme-suite.org) with the following parameters: the number of different motifs set as 10, and the minimum motif and maximum motif windows set to 6 and 50, respectively. We determined the identity and functions of Ta*ARF* conserved domains by using their protein sequences as query for the SMART (http://smart.embl-heidelberg.de/) and Pfam databases (http://pfam.sanger.ac.uk/).

### Chromosomal Distribution and Gene Duplication of *TaARF* Genes

We assigned each *TaARF* gene to its designated chromosome by investigating their chromosomal positions provided by the Ensembl Plants database. We investigated *TaARF* gene duplication events by pairwise alignments based on previously used criteria with minor adjustment: (a) the pairwise alignment should cover more than 80% of the longer gene; (b) the percentage identity in the aligned region should be higher than 70%; and (c) only one duplication event was counted for tightly linked genes (Gill et al., 2004; Wang et al., 2016). The chromosomal distribution, gene duplication events, and homoeologous copies of *TaARF* genes were visualized along the *T. aestivum* genome using Circos version 0.69 (http://circos.ca/).

### Predicted Small RNAs Targets and Putative Untranslated ORF Detection

We downloaded the sequences of wheat small RNAs from miRbase (http://www.mirbase.org/), combined with results from our previous work (Tang et al., 2012). Small RNA targets were identified by The psRNATarget Schema V2 server (http://plantgrn.noble.org/psRNATarget/home) and TAPIR (http://bioinformatics.psb.ugent.be/webtools/tapir/) set to the default value of maximum expectation of 3.5 for compatibility reasons, and results that overlapped with targets of small RNAs identiﬁed by degradome sequencing in wheat in a previous study (Tang et al., 2012) were considered credible. We extracted *TaARF* 5′ untranslated regions (UTRs) from the Ensembl Plants database. Potential untranslated open reading frames (uORFs) were examined by ORF Finder (http://www.geneinfinity.org/sms/sms_orffinder.html).

### Promoter Sequence Analysis

We retrieved 2 kb of genomic DNA sequences upstream of the initiation codon ATG for each *TaARF* gene from the International Wheat Genome Sequencing Consortium (IWGSC) genome assembly. The promoter sequences were used to query the wheat protein database by BLASTX (translated nucleotide against protein database) to confirm that none contained protein-coding sequences. To search for *cis*-regulatory elements associated with abiotic stress and hormone-dependent responses within promoter regions, we submitted the promoter sequences to the PLACE database (https://www.dna.affrc.go.jp/PLACE/?action=newplace).

### Plant Materials and Stress Treatments

We harvested from field-grown wheat plants (*Triticum aestivum* cv. YM12) at growth stage GS51: roots, stems, leaves, spikelets (anthers removed), and anthers at different developmental stages (S7: meiotic stage anther, S9: young microspore stage anther, S12: 3-nucleate pollen stage anther) (Browne et al., 2018). For abiotic stress treatments, we used seeds from the wheat cultivar Jinghua 9, and exposed 14-day-old seedlings to drought (incubation in 25% (w/v) PEG-6000), cold (grown at 4°C), or salt (incubation in 250 mM NaCl) stress and abscisic acid (ABA) treatment (spraying with 0.1 mM ABA) for 0, 1, 2, 5, 10, or 24 h as described (Xu et al., 2016). Seedlings that were not exposed to these respective treatments served as controls. At stipulated times, the seedlings were immediately frozen in liquid nitrogen and stored at −80°C.

The fertility of the TGMS wheat line BS366 is controlled by temperature (Tang et al., 2011). We also used the two wheat TGMS lines BS366 and BS1088 (selected from a natural mutant of doubled haploid lines) and one common wheat variety J411, from our laboratory seed stocks. The plants were grown in phytotrons at 20°C under a 12-h light/12-h dark photoperiod during the entire reproductive period. We initiated low-temperature treatments (consisting of 10°C under a 12-h light/12-h dark photoperiod) at the stamen primordia stage (when half of the ﬂag leaf has emerged from the collar of the penultimate leaf). We collected anthers at different developmental stages (S6: stage 6, central callose stage; S7: stage 7, meiotic stage; S8: stage 8, tetrad stage; S9: stage 9, young microspore stage) from plants exposed to cold or maintained in control conditions, as described previously (Tang et al., 2011). All samples were immediately frozen in liquid nitrogen and stored at –80°C. Spikelets were harvested before anthesis and ﬁxed with 3:1 ethanol:acetic acid (v/v). The pollen was stained with 2% iodine-potassium iodide.

### RNA Isolation and RT-qPCR Analysis

We extracted total RNA using TRIzol reagent according to manufacturer’s instructions. For each sample, 1 µg of total RNA was treated with RNase-free DNase I (TaKaRa, Dalian, China) to eliminate genomic DNA contamination. First-strand cDNA was synthesized with an oligodT_24_ primer and TaKaRa PrimeScript RT Reagent Kit (TaKaRa, Dalian, China). We then determined the relative transcript levels of genes of interest with TaKaRa SYBR Premix Ex Taq (Tli RNase H Plus) (TaKaRa, Dalian, China) on a Eco Real-Time PCR System (Illumina). Each sample was collected as three independent biological replicates, each consisting of three technical replicates. The wheat *ACTIN* gene (GenBank accession: 542814) was used as an internal control.

Redundant and gene-specific primers were designed to amplify the homoeologous copies of each *TaARF* gene from the A, B, and/or D subgenomes using Primer Premier 5.0 (**Supplementary Table S6**). We performed RT-qPCR reactions with the following parameters: 30 s at 94°C to start, followed by 40 cycles of 95°C for 5 s, 58°C for 30 s. Then, a melting curve of 95°C for 15 s, 60°C for 60 s, and 95°C for 15 sec was used. The relative gene expression levels were analyzed according to the 2^−ΔΔCT^ method.

## Results

### Identification of *ARF* Family Genes From *T. aestivum*, *T. urartu*, *A. tauschii*, *T. turgidum*, and *H. vulgare*

To identify *ARF* genes in *T. aestivum*, *T. urartu*, *A. tauschii*, *T. turgidum*, and *H. vulgare*, we searched their genomes at the Ensembl Plants database using the Basic Local Alignment Tool for Protein (BLASTP) with 23 *Arabidopsis* ARF and 25 rice ARF proteins as queries. We looked for conserved ARF domains by HMMER in all candidates, and excluded any gene encoding truncated ARF proteins. We thus identified 69 *TaARF* members from the wheat A, B, and D subgenomes and assigned them to 24 homoeologous groups.

In addition, we identified the complement of *ARF* genes from *T. urartu*, *A. tauschii*, and *H. vulgare* following the same procedure: we discovered 19 full-length *ARF* genes in *T. urartu*, 23 in *A. tauschii*, 43 in *T. turgidum*, and 22 in *H. vulgare*, in addition to one truncated gene in *T. urartu* (*TuARF5*), *T. turgidum* (*TtARF5-A*), and *H. vulgare* (*HvARF8*) (**Supplementary Table S1**). We numbered *TaARF* genes from *TaARF1* to *TaARF24* according to their chromosomal locations, while *TuARF*, *AeARF*, *TtARF*, and *HvARF* genes were given the same names as their wheat orthologs (**Supplementary Table S1**).

The predicted molecular weights of the identified ARF proteins varied greatly, ranging from 38.8 to 130.9 kDa (*T. aestivum*), 28.0 to 137.5 kDa (*T. urartu*), 38.8 to 128.7 kDa (*A. tauschii*), 25.90 to 126.31 kDa (*T. turgidum*), and 50.3 to 125.4 kDa (*H. vulgare*). Similarly, their predicted isoelectric points (pI) pointed to both acidic and basic ARF proteins, with pIs ranging from 5.29 to 7.98 (*T. aestivum*), 5.61 to 8.95 (*T. urartu*), 5.47 to 8.54 (*A. tauschii*), 5.79 to 9.46 (*T. turgidum*), and 5.74 to 8.21 (*H. vulgare*). Most TaARF proteins were predicted to be nuclear proteins, with the exception of TaARF1 and TaARF8 (chloroplast), TaARF23 (cytosol), and TaARF24 (mitochondria) (**Supplementary Table S1**).

### Phylogenetic Analysis of *ARF* Genes in Wheat

**Figure 1:**
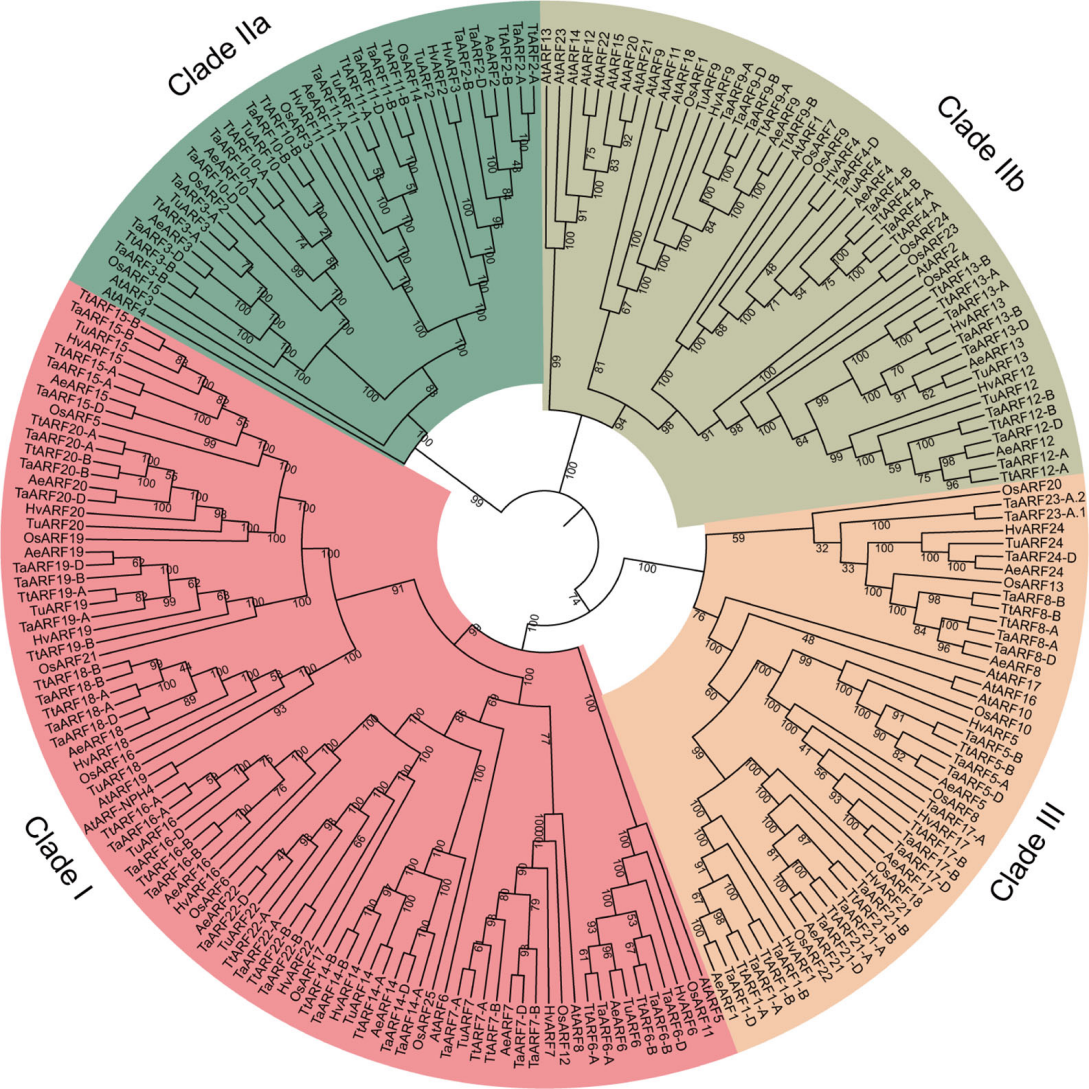
Phylogenetic analysis of the ARF family in wheat and related species. We aligned 221 ARF proteins from *T. aestivum* (*Ta*), *T. urartu* (*Tu*), *A. tauschii* (*Ae*), *T. turgidum* (*Tt*), *H. vulgare* (*Hv*), *O. sativa* (*Os*), and *A. thaliana* (*At*) with MUSCLE and drew the phylogenetic tree in MEGA 7 with the maximum-likelihood method. The tree was classified into three clades (Clade I, Clade IIa and IIb, and Clade III). Confidence values are listed at each node.

To elucidate the phylogenetic relationship of *ARF* genes across species, we constructed a maximum-likelihood phylogenetic tree using the protein sequences of 69 TaARFs, 18 TuARFs, 23 AeARFs, 42 TtARFs, and 21 HvARFs identified in this study, in addition to 25 rice ARFs (OsARFs) and 23 *Arabidopsis* ARFs (AtARFs) found in previous studies. We excluded truncated proteins. Our phylogenetic analysis revealed three distinct and evolutionarily conserved ARF clades (**Figure 1**), consistent with previous results obtained from the ARF family in *Arabidopsis* and rice (Okushima et al., 2005; Finet et al., 2013). We divided Clade II into two subgroups, IIa and IIb. Based on reporter gene assays (Ulmasov et al., 1999a; Tiwari et al., 2003), Clade I ARFs are thought to be transcriptional activators, whereas Clade IIb ARFs are generally considered to be repressors. Clade IIa ARFs include *Arabidopsis* ARF3, also named ETTIN; Clade III ARFs do not affect reporter gene expression. The phylogenetic tree showed that 86 members clustered into Clade I (with 9 TaARFs). Clade IIa included 37 members (with 4 TaARFs), and Clade IIb had 51 members (with 4 TaARFs). Clade III contained 47 members (with 7 TaARFs) (**Figure 1**).

Most *TaARF* genes had clear orthologs in the progenitor genomes, from the A genome lineage (*T. urartu*), D lineage (*A. tauschii*), and AABB lineage (*T. turgidum*). Only four *TaARF* genes (*TaARF1*, *TaARF8*, *TaARF17*, and *TaARF21*) lacked a clear ortholog in the A genome lineage (*T. urartu*) (**Figure 1** and **Supplementary Table S1**). In addition, *TaARF23* had no obvious homoeolog in the wheat B or D subgenomes, just as it lacked a clear ortholog in the A, D, and AABB lineages. Likewise, *TaARF24* had no homoeolog in the A or B wheat subgenomes, and lacked a clear ortholog in the AABB lineage (**Figure 1** and **Supplementary Table S1**).

To understand the evolutionary processes shaping the *ARF* gene family in *T. aestivum*, *T. urartu*, *A. tauschii*, *T. turgidum*, and *H. vulgare*, we investigated the degree of genetic differentiation and selective pressure experienced by *ARF* genes within the five species using DnaSP 5.0. Nucleotide diversity (Pi) for *ARF* genes between the five species was 0.355 (*T. aestivum*), 0.427 (*T. urartu*), 0.407 (*A. tauschii*), 0.448 (*T. turgidum*), and 0.403 (*H. vulgare*), values that were clearly higher those measured for each *ARF* gene across the different species (**Supplementary Table S2**). Indeed, nucleotide diversity for individual *ARF* genes between the five species *T. aestivum*, *T. urartu*, *A. tauschii*, *T. turgidum*, and *H. vulgare* was very low, except in the case of *ARF24*, for which the Pi value was two to eight times higher than for any other *ARF* gene (**Supplementary Table S2**). These results indicated that individual *ARF* genes were highly conserved between the five species (had low Pi value), whereas the *ARF* gene family from each species showed more diversity with respect to those of other species (high Pi value). We next performed an analysis of Tajima’s *D* to determine whether *ARF* genes have been under selective pressure among the five species. No *ARF* gene showed a significant departure from the neutral expectation (**Supplementary Table S2**), indicating that no selective pressure operated on *ARF* genes during their evolution.

### Structure Analysis and Genomic Distribution of *TaARF* Genes

**Figure 2:**
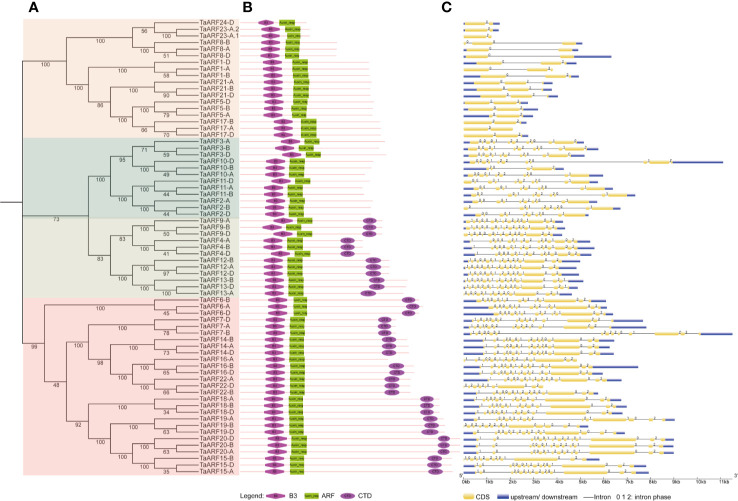
Phylogenetic relationships and gene structure of *ARF* gene family members in wheat. **(A)** Phylogenetic tree of wheat ARF proteins, constructed in MEGA 7.0 with the neighbor-joining (NJ) method. **(B)** Conserved domains in ARF proteins, identified by the SMART database, represented by different shapes and colors. Pink diamond: B3 domain; green rectangle: ARF domain; purple oval: CTD domain. **(C)** Gene structure of *TaARF* genes. Gene features were extracted from the Ensembl Plants database and displayed by GSDS (http://gsds.cbi.pku.edu.cn/). Yellow indicates exons, blue indicates 5′ and 3′ UTRs, black lines indicate introns, and the intron phase is displayed above each intron.

The analysis of the number and position of exons, introns, and conserved elements provides a foundation for our understanding of gene structural evolution, and thus function. We observed that all TaARFs have a B3 domain and an ARF domain, and most TaARFs also harbor a conserved CTD domain, with the exception of those in Clades IIa and III (**Figure 2B**). An analysis of exon–intron organization structure revealed that *TaARF* genes contained 12–15 exons, with 11–14 introns in Clade I, 10–14 exons and 9–13 introns in Clade II, and 2–3 exons and 1–2 introns in Clade III (**Figure 2C**).

The domain composition of TaARF proteins and structure of *TaARF* genes from paralogous groups are well conserved, including symmetric exons and associated intron phases (**Figure 2**). We identified all functional protein domains in TaARFs *via* the SMART database. We searched for all functional domains within ARF proteins with the MEME suite web server: of ten motifs detected, eight comprised the typical ARF domains. Indeed, the B3 domain corresponded to motifs 2, 3, and 4; the ARF domain was composed of motifs 6, 7, and 8; and motifs 9 and 10 formed the CTD domain (**Supplementary Figure S1**).

**Figure 3:**
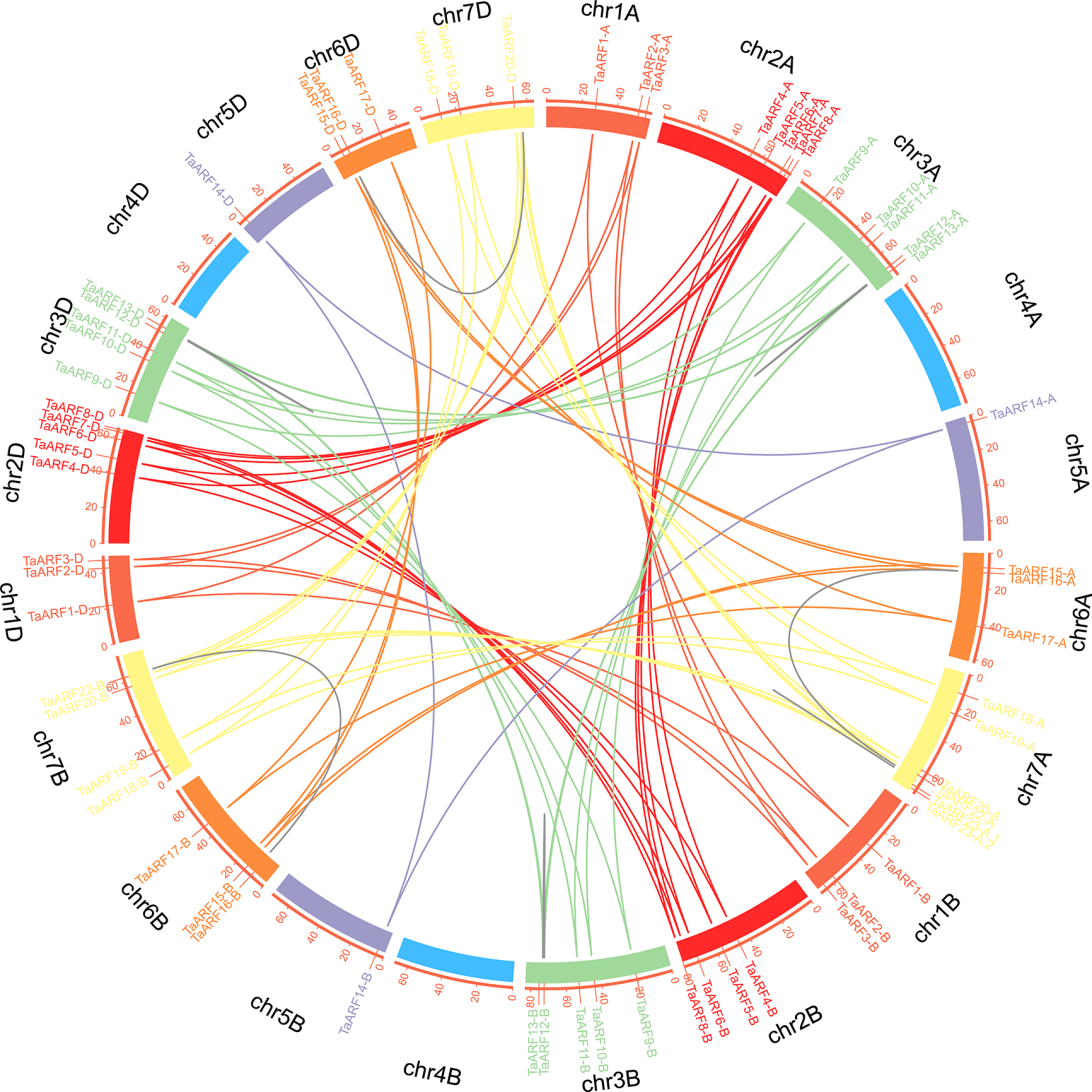
Chromosomal localization and duplication events of *TaARF* genes in wheat. Each *TaARF* gene is indicated by a vertical red line on the outer-most circle. Seven *TaARF* homoeologous chromosome groups are represented in different colors, and homoeologous *TaARF* copies are linked by lines of the same color. Duplicated *TaARF* gene pairs are connected by grey lines. Note the four tightly linked gene pairs on chr3A, chr7A, chr3B, and chr3D.

We then built a chromosomal map to determine the chromosomal distribution of all wheat *ARF* genes. We mapped 69 *TaARF* genes onto six of the seven wheat chromosomes (chromosome 4 the only one not containing a single *ARF* gene) (**Figure 3**). Across all three subgenomes, chromosome group 7 had the most *ARF* genes (18, or ~26.1%), followed by chromosome groups 2 and 3 (15 each, or ~21.7%). Chromosome groups 6 (9 genes, ~13.0%), 1 (9 genes, ~13.0%), and 5 (3 genes, ~4.3%) rounded off the remaining *ARF* genes. In agreement with our previous identification of homoeologous groups, sub-genome A included 24 *TaARF* genes, followed by sub-genome D with 23 *TaARF* genes, and sub-genome B with 22 *TaARF* genes. In addition, we determined that three pairs of paralogous groups represented duplication events, including two tightly linked duplication events (*TaARF12* and *TaARF13* on chromosome 3A and *TaARF23-A.1* and *TaARF23-A.2* on chromosome 7A) and one case of segmental duplication event between two chromosomes (*TaARF16* on chromosome 6A and *TaARF22* on chromosome 7A). These pairs of duplicated genes shared 80.6% (*TaARF12* and *TaARF13*), 99.2% (*TaARF23-A.1* and *TaARF23-A.2*), and 77% (*TaARF16* and *TaARF22*) nucleotide sequence identity (**Figure 3** and **Supplementary Table S3**). This high degree of sequence identity is suggestive of functional redundancy.

### Prediction of Potential Regulators of *TaARF* Transcript Levels: Small Regulatory RNA and Upstream ORFs

MicroRNAs (miRNAs) play pivotal roles in the regulation of transcript levels and are involved in numerous developmental processes in plants (D’Ario et al., 2017). There is a growing body of evidence supporting the existence of small-RNA-mediated regulation of *ARF* transcript levels by direct cleavage at a site in the target *ARF* mRNA that is complementary to the miRNA. For example, in *Arabidopsis*, *ARF2*, *ARF3*, and *ARF4* are inhibited by tasiRNAs that regulate *ARF* genes (tasiR-ARFs) (Marin et al., 2010).

To investigate the potential regulation of *TaARF* genes by miRNAs, we used published wheat small RNA datasets to determine whether any would target *TaARF* genes. In our phylogenetic tree, *TaARF2*, *TaARF3*, *TaARF10*, and *TaARF11* all clustered with *Arabidopsis ARF3* within Clade IIa and all four *TaARF* genes were predicted to be targets for the wheat tasiRNA-ARFs (**Supplementary Table S4**), which suggests that the miR390–*TRANS-ACTING SIRNA3* (*TAS3)*–*ARF* regulation module may be conserved in wheat; miR390 promotes the production of tasiRNAs from *TAS3* transcripts, which in turn regulate *ARF* transcript levels. In addition, *Arabidopsis* miR167 targets *ARF6* and *ARF8*, while *ARF10*, *ARF16*, and *ARF17* are targets of miR160 (Wu et al., 2006; Liu et al., 2007). We predicted that the wheat *ARF* genes *TaARF1* and *TaARF17* contained target sites for tae-miR160, whereas *TaARF7* is targeted by tae-miR167, and *TaARF14* and *TaARF16* are putative targets for tae-miR167d (**Supplementary Table S4**). All miR160 targets in *Arabidopsis* and wheat belonged to class III and were considered as potential orthologs, whereas miR167 targeted *ARF* genes from Clade I (**Figure 1**), underscoring the conserved miRNA target sites and common mechanism of gene expression regulation in dicots and monocots.

Upstream open reading frames (uORFs) are *cis*-elements that are located upstream of the main coding region and modulate translation initiation efficiency of the downstream main ORF (Zouine et al., 2014; Spealman et al., 2018). Ribosome profiling studies have shown that a number of uORFs initiate with non-AUG start codons, which are translated less efficiently than AUG-containing uORFs (Spealman et al., 2018). We searched *TaARF* 5′ untranslated regions to detect putative uORFs: 40 out of 69 *TaARF* genes harbored non-AUG uORFs (initiating from non-AUG start codons), ranging from 1 to 5 per gene, while AUG uORFs were observed in 11 *TaARF* genes, with a range of 1–3 per gene (**Supplementary Table S5**). Therefore, uORFs may play important roles in regulating *TaARF* expression.

### Identiﬁcation of *cis*-Elements in *TaARF* Promoters

**Figure 4:**
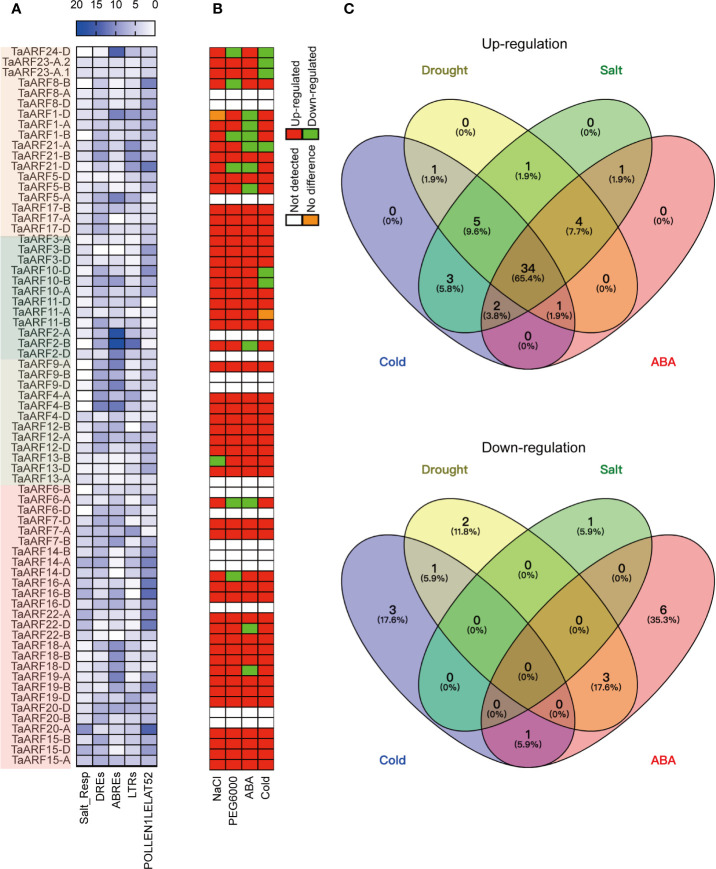
Response of *TaARF* transcript levels to drought (PEG6000), cold, NaCl and ABA treatment. **(A)** Numbers of *cis*-elements identified in *TaARF* promoters. *TaARF* genes are sorted according to the phylogenetic tree shown in **Figure 1**, with each clade indicated by a different color. **(B)** Summary representation of *TaARF* up- and down-regulation in response to stress based on RT-qPCR. Red: up-regulated; green: down-regulated; orange: no difference; white: not detected. **(C)** Venn diagram for up- (top) and down- (bottom) regulated *TaARF* genes after exposure to different abiotic stresses or ABA treatment. Each section of the diagrams shows genes that are speciﬁc for a single stress (salt, drought, cold, or ABA) or that are shared across multiple conditions.

To gain a better understanding of the dynamic regulation of *TaARF* gene expression in response to abiotic stresses and during plant growth, we analyzed the occurrence and distribution of *cis*-regulatory elements within a 2-kb genomic region upstream of the transcription start site. We estimated the number and positions of several *cis*-regulatory elements known to be involved in phytohormone responses, as well as response to abiotic stresses (**Figure 4A** and **Supplementary Figure S2**). These phytohormone *cis*-regulatory elements included auxin-responsive elements (AUXRETGA2GMGH3, AUXREPSIAA4, and NTBBF1ARROLB), methyl jasmonate (MeJA)-responsive elements (T/GBOXATPIN2), ethylene-responsive elements (GCCCORE), salicylic-acid-responsive elements (ASF1MOTIFCAMV and WBOXATNPR1), and a gibberellin-responsive element (GAREAT; **Supplementary Figure S2**). This list of *cis*-regulatory elements was similar to those seen in barrel clover (*Medicago truncatula*) and sweet orange (*Citrus × sinensis*) (Li et al., 2015; Shen et al., 2015), suggesting that *TaARF* genes may be involved in phytohormone-mediated regulation of signal transduction processes.

In addition, over-represented abiotic-stress-related *cis*-regulatory elements observed in the promoters of *TaARF* genes included a salt-responsive element (Salt_Resp: GT1GMSCAM4), dehydration-responsive elements (DREs: DPBFCOREDCDC3 and DRECRTCOREAT), and ABA-responsive elements (ABREs: ABREATCONSENSUS, ABREOSRAB21, ABREATRD22, and ABRELATERD1), low-temperature-responsive elements (LTRs: LTREATLTI78, LTRECOREATCOR15, and LTRE1HVBLT49) (**Figure 4A** and **Supplementary Figure S2**). Moreover, a subset of *TaARF* promoters contained the ARFAT element (auxin response factor binding site), whereas the pollen-specific element POLLEN1LELAT52 was observed in all *TaARF* genes, with the exception of *TaARF11-D* (**Figure 4A**). These results suggest that *TaARF* genes may be involved in regulating a variety of stress responses and preferentially expressed in individual tissues.

### Expression of *TaARF* Homoeologs in Response to Abiotic Stress

Extensive studies have revealed that the expression of *ARF* genes responds to abiotic stress. We therefore performed RT-qPCR analysis to determine *TaARF* gene expression levels in seedlings treated with NaCl, PEG6000, cold, and ABA to mimic salt, drought, and cold stress and phytohormone stimulation, respectively. Out of 69 *TaARF* genes, 15 showed very low or undetectable transcript levels at the seedling stage; for three (*TaARF9B*, *TaARF20-B*, and *TaARF20-D*), we were unable to design effective gene-specific primers. The expression level of the remaining 51 *TaARF* genes from 24 homoeologous groups rose above the detection level and exhibited widely divergent expression patterns. Using a standard of 2-fold change (either up- or down-regulated) with respect to control seedlings, 23 of 24 *TaARF* homoeologous groups were induced in response to NaCl treatment, the sole exception being *TaARF13-B*, which was repressed by NaCl treatment (**Figure 4B**). The majority of *TaARF* genes exhibited a peak in expression levels after 10 h of NaCl exposure. Five *TaARF* genes reached their highest expression levels within 1–2 h and subsequently gradually decreased (**Supplementary Figure S3**).

*TaARF* genes generally responded very early to PEG6000 treatment. Within 1 h of PEG6000 treatment, 45 *TaARF* genes from 19 homoeologous groups reached their highest expression levels, followed by a gradual decrease back to, or to even lower than, their expression levels at time 0. The remaining 6 detectable genes were down-regulated by PEG6000 treatment relative to the control (**Figure 4B** and **Supplementary Figure S4**). Treatment with the phytohormone ABA raised transcript levels for 42 *TaARF* genes assigned to 21 homoeologous groups after 5 or 10 h of treatment, a later response than when plants were treated with PEG6000. The transcript levels for most *TaARF* genes returned to control levels by 24 h (**Supplementary Figure S5**). In addition, another 9 *TaARF* genes belonging to 7 homoeologous groups were down-regulated by ABA treatment (**Figure 4B**). Under cold stress, *TaARF* genes responded with similar kinetics: 46 *TaARF* genes were upregulated, and the remaining 5 were down-regulated (**Supplementary Figure S6**). A Venn diagram analysis revealed that 39 *TaARF* genes were up-regulated in response to both PEG6000 and ABA treatment, while another three *TaARF* genes were down-regulated by both treatments. In addition, 34 *TaARF* genes belonging to 15 homoeologous groups (*TaARF3*, *TaARF4*, *TaARF5*, *TaARF9*, *TaARF10*, *TaARF11*, *TaARF12*, *TaARF13*, *TaARF15*, *TaARF17*, *TaARF18*, *TaARF19*, *TaARF20*, *TaARF21*, and *TaARF22*) were up-regulated in response to all four treatments (NaCl, PEG6000, cold stress, and ABA), while several *TaARF* genes responded only to an individual stress (**Figures 4B, C**).

These results also provided the basis for gain an understanding of the similarities and divergence between homoeologs in the context of their expression patterns in seedlings during abiotic stresses and ABA treatment. *TaARF23* and *TaARF24* each have only a single homoeolog and three *TaARFs* unable to design effective gene-specific primers, and thus we focused on the 60 *TaARF* genes that were assigned to 20 complete homoeologous groups (with one gene per sub-genome). According to a previous study (Ramírez-González et al., 2018), expression of homoeologous groups (or here, triads) can follow one of three homoeolog expression bias categories: balanced category, with similar relative expression levels for all three homoeologs; homoeolog-dominant category, considering the most highly expressed homoeolog; homoeolog-suppressed category, considering the least expressed homoeolog.

In our data, most syntenic triads showed single-homoeolog-dominant expression patterns in seedlings during abiotic stress and ABA treatment (**Figures 6A–D**). In response to low temperature, 11 of the 20 triads were single-homoeolog dominant. *TaARF3* was the only case of single-homoeolog suppression, whereas *TaARF1*, *TaARF11*, *TaARF12*, *TaARF15*, *TaARF18*, *TaARF19*, *TaARF21*, and *TaARF22* were assigned to the balanced category. Looking at single stresses, 13 triads of *TaARF* genes were examples of single-homoeolog dominance under NaCl treatment, and *TaARF1*, *TaARF5*, and *TaARF16* were single-homoeolog-suppressed under the same conditions, with the remaining 4 triads belonging to the balanced category. After treatment with PEG6000 and ABA, 6 *TaARF* triads showed similar expression patterns, whereas 10 triads were examples of single-homoeolog dominance. Finally, four *TaARF* triads (*TaARF5*, *TaARF16*, *TaARF18*, and *TaARF21*) showed single-homoeolog suppression upon both PEG6000 and ABA treatment. These results indicate *TaARF* members of the homoeologous triads show a mixture of conserved and divergent expression under abiotic stress and ABA treatment.

### Expression Patterns of *TaARF* Genes in Different Tissues

**Figure 5:**
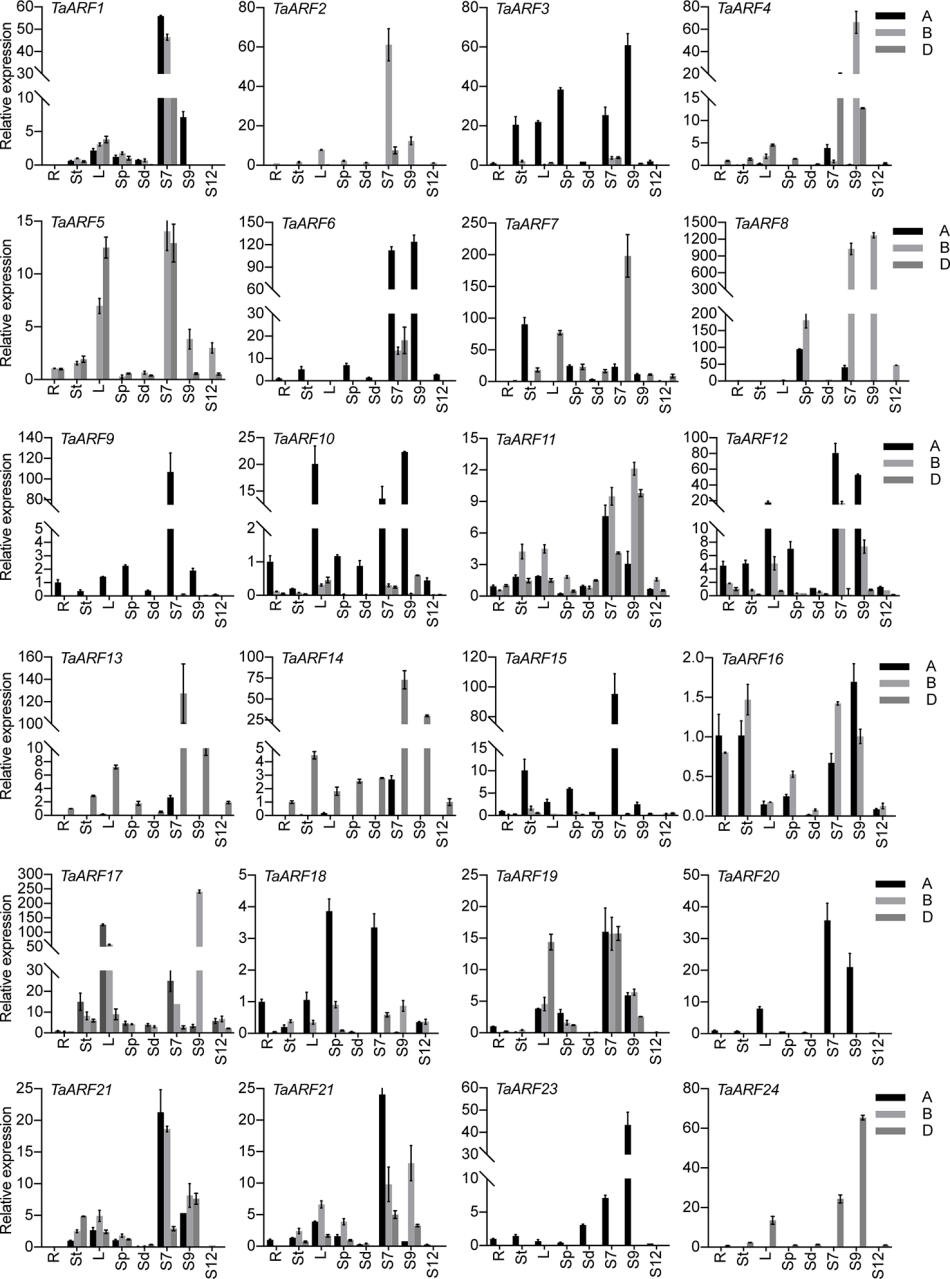
Expression patterns of *TaARF* genes in various tissues. R: root; St: stem; L: leaves; Sp: spike (anther removed); Sd: seed; S7: stage 7, anther at meiotic stage; S9: stage 9, anther at young microspore stage; S12: stage 12, anther at 3-nucleate pollen stage. Data represent the mean of three biological repeats; error bars represent the standard error.

To probe the physiological roles of *TaARF* genes, we conducted an RT-qPCR analysis of *TaARF* gene expression profiles in eight different vegetative and reproductive tissues (root, stem, leaves, spike, seeds, and anthers at three different stages). We observed *TaARF* gene expression in all plant developmental stages under normal growth conditions and identified a set of *TaARFs* that were preferentially expressed in a particular tissue rather than in other organs (a >2-fold change with respect to expression in all other organs). Most *TaARF* genes were up-regulated during anther development (**Figure 5**). We detected preferential expression in anthers for 17 *TaARF* triads (*TaARF1*, *TaARF2*, *TaARF4*, *TaARF6*, *TaARF7*, *TaARF8*, *TaARF9*, *TaARF11*, *TaARF12, TaARF13*, *TaARF14*, *TaARF15*, *TaARF20*, *TaARF21*, *TaARF22*, *TaARF23*, and *TaARF24*) at stage 7 and stage 9, but no *TaARF* gene showed high transcript levels at stage 12. Among 17 anther-enriched *TaARF* genes, *TaARF6*, *TaARF7*, *TaARF9*, and *TaARF13* displayed a 106- to 198-fold increase in expression in anthers relative to their expression in roots, with as high as a 1,269-fold rise for *TaARF8*. By contrast, *TaARF5*, *TaARF10*, *TaARF17*, and *TaARF19* were more highly expressed in leaves and anthers than in roots. The genes *TaARF3*, *TaARF16*, and *TaARF18* were expressed ubiquitously in all tissues.

**Figure 6:**
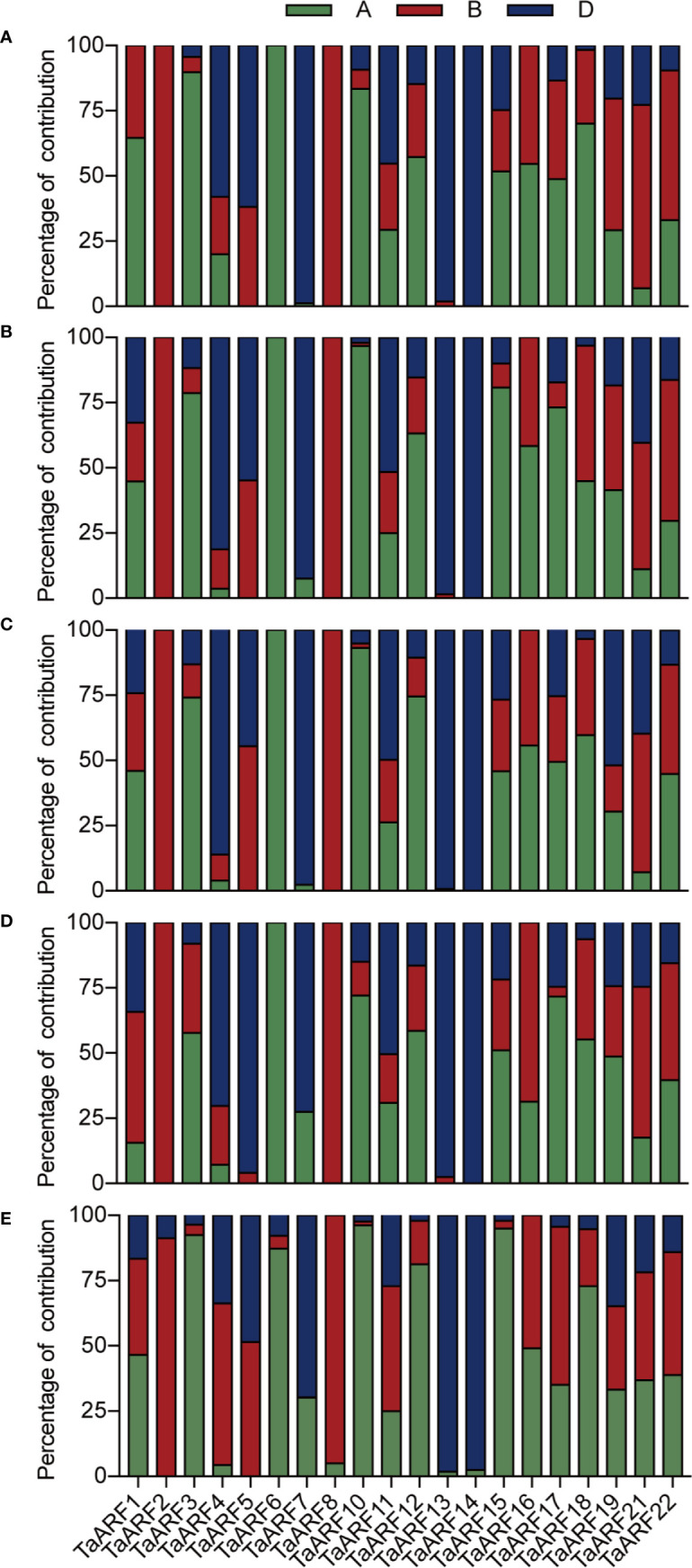
The relative contribution of the A, B, and D genome homoeolog *TaARF* to the overall triad expression across NaCl **(A)**, PEG6000 **(B)**, ABA **(C)**, cold treatment **(D)**, and all tissues **(E)** based on RT-qPCR. The relative expression level of the three homoeologs was added, and this was used as the denominator to obtain the normalized triad expression value for each gene/triad.

We observed homoeolog expression bias among syntenic homoeolog triads across all tissues (**Figure 6E**). Among 17 *TaARF* genes preferentially expressed in anthers, *TaARF1*, *TaARF11*, *TaARF21*, and *TaARF22* homoeologs shared similar expression levels, while *TaARF7* showed single-homoeolog suppression and the remaining 8 triads were classified as cases of single-homoeolog dominance. The ubiquitously expressed *TaARF* genes (*TaARF3* and *TaARF18*), along with *TaARF10* (highly expressed in leaves) showed single-homoeolog dominance, whereas *TaARF19* was assigned to the balanced category. The remaining *TaARF5*, *TaARF16*, and *TaARF17* showed single-homoeolog suppression. Thus, from these analyses, we inferred the relative contribution of each *TaARF* homoeolog to the overall triad expression across all tissues to help us to distinguish whether gene homoeologs are functionally redundant or showing dominance effects.

### Expression Profiles of Anther-Preferential *TaARF* Genes in TGMS Wheat Lines

**Figure 7:**
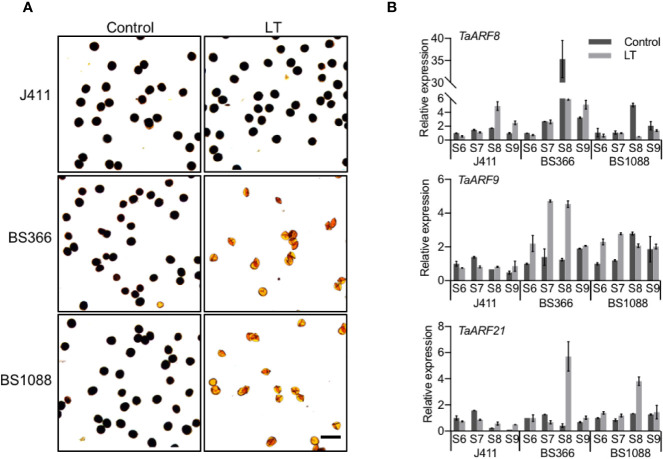
Anther-preferential *TaARF* genes associated with cold-induced male fertility. **(A)** Morphology of pollen grains in TGMS wheat and common wheat variety J411. At low temperature (LT, 10°C under a 12-h light/12-h dark photoperiod) and control conditions (control, 20°C under a 12-h light/12-h dark photoperiod), pollen grains from the two male sterile lines BS366 and BS1088 anther are devoid of starch (stained with Lugol’s iodine). Bars = 100 µm. **(B)** Expression pattern of male-sterility-related *TaARF* genes in anthers of the TGMS lines exposed to low temperature (LT), compared with anthers from control plants (Control). S6: stage 6, central callose stage; S7: stage 7, meiotic stage; S8: stage 8, tetrad stage; S9: stage 9, young microspore stage.

To explore the potential functions of *TaARF* genes specifically expressed in anthers, we measured their transcript levels in TGMS wheat lines during male reproduction. Exposure of TGMS wheat lines to low temperature, beginning at stage 6 and continuing to the end of stage 9, results in shrunken anthers and starch-deficient pollen grains (Tang et al., 2011; **Figure 7A**). However, these lines are fully male-fertile with starch-filled normal pollen when grown under control conditions, suggesting that genes responsible for the TGMS phenotype may themselves be highly expressed between the stage 6 and stage 9 of anther development. Common wheat variety J411 with low temperature treatment was choose as a control for comparison with the cold-treated TGMS wheat. Thus, we could identify genes that play crucial roles in anther development during exposure to cold stress in the TGMS wheat, and discount genes that are only responding to cold-regulated pathways.

We checked the expression of 17 *TaARF* genes that were preferentially expressed in anthers between anther stages 6 and stage 9 in the TGMS line BS366 under low-temperature and control conditions by RT-qPCR. In hexaploid wheat, since most gene homoeologs are highly similar and may act redundantly, we designed PCR primers that amplified all three homoeologs. In TGMS line BS366, three *TaARF* triads (*TaARF6*, *TaARF9*, *TaARF11*, *TaARF12*, and *TaARF14*) exhibited higher transcript abundance during anther development under low temperatures, with a fold change of at least 2, whereas five triads (*TaARF8*, *TaARF13*, *TaARF20*, *TaARF23*, and *TaARF24*) were particularly highly expressed in control conditions. In addition, the expression level of the *TaARF1*, *TaARF15*, *TaARF21*, and *TaARF22* triads rose steadily after an initial drop during the stage 6 at low temperatures (**Supplementary Figure S7**). The remaining three genes (*TaARF2*, *TaARF4*, and *TaARF7*) showed no significant change in expression (**Supplementary Figure S7**). Looking at *TaARF* gene expression profiles in more detail, *TaARF6*, *TaARF9*, *TaARF14*, and *TaARF22* were up-regulated 2- to 5-fold with respect to the control from the stage 7 to stage 9 under low temperature. *TaARF23* showed a nearly identical expression pattern as *TaARF24* when exposed to low temperature, with reduced expression from stage 7 to stage 9. The expression of *TaARF8* and *TaARF13* decreased 5- to 6-fold at stage 8 at low temperatures, while *TaARF20* was down-regulated specifically at stage 9.

The expression of 14 *TaARF* genes exhibited at least 2-fold change at low temperature when compared to that in control conditions in the BS366 line. We next sought to further confirm their expression profiles in the other TGMS line BS1088 (**Supplementary Figure S8**). Only three *TaARF* genes were differentially expressed in the BS1088 line (**Figure 7B**). *TaARF8* was down-regulated to very low transcript abundance at stage 8 in low temperature with respect to the control in BS1088, while the expression of *TaARF8* in the cold-treated J411 controls showed the reverse pattern. Interestingly, *TaARF9* exhibited high levels of transcript at stage 6 and stage 7 in low temperature in BS1088, but no signiﬁcant changes in the cold-treated J411 controls. *TaARF21* was highly expressed at stage 8 in both BS1088 and J411 under low temperature, suggesting that *TaARF21* only responds to cold-regulated pathways. Overall, *TaARF8* and *TaARF9* exhibited different expression trends during anther development in TGMS lines and ordinary wheat, indicating that *TaARF8* and *TaARF9* respond to cold in anthers of the TGMS lines, marking them as potential components of the for TGMS mechanism in wheat.

## Discussion

### The *ARF* Gene Family in Hexaploid Bread Wheat and Its Progenitors

*ARF* genes are a class of plant-specific transcription factors (B3-type) that regulate the expression of auxin response genes. The *ARF* gene family has been extensively described in flowering plants, such as *Arabidopsis*, rice, soybean, and maize (*Zea mays*) (Li et al., 2016). However, our knowledge about the ARF protein family in Triticeae species such as wheat is very limited. After two polyploidization events, the combined genomes of hexaploid bread wheat (AABBDD) have almost triple the number of genes in their diploid wheat progenitors (AA or DD) (The International Wheat Genome Sequencing Consortium (IWGSC) et al., 2014). In this study, we identified 24, 22, and 23 *TaARF* genes in the bread wheat A, B, and D sub-genomes, respectively. Comparing the sizes of the *ARF* gene family in the *A. tauschii* and *T. urartu* diploid genomes and the *T. turgidum* tetraploid genome with those in the individual subgenomes of hexaploid bread wheat, we were able to match 69 *TaARF* members with clear orthologs in the diploid wheat progenitors (23 in *A. tauschii* and 19 in *T. urartu*), and only *TaARF23* and *TaARF24* lacked an ortholog in tetraploid wheat (*T. turgidum*) (**Supplementary Table S1**). Furthermore, five genes (*TaARF1*, *TaARF8*, *TaARF17*, *TaARF21*, and *TaARF23*) had no ortholog in the A genome lineage (*T. urartu*) and/or the D lineage (*A. tauschii*) and thus may have originated from gene expansion after polyploidization or may represent incomplete sequence coverage or assembly errors (The International Wheat Genome Sequencing Consortium (IWGSC) et al., 2014). Likewise, *TaARF24* has apparently lost the homoeologous copies derived from the A and B subgenomes, suggestive of either gene loss after polyploidization or incomplete coverage in the wheat reference genome.

### Phylogeny and Structural Characteristics of ARF Proteins in Wheat

Phylogenetic analysis of ARF proteins provided insight into the evolutionary relationships of family members across species as well as putative functional assignments. Most *ARF* genes possess a conserved structure and a similar function since the divergence between monocots and dicots (Guilfoyle and Hagen, 2007). Thus, based on our phylogenetic analysis, we explored the phylogenetic relationships of ARF proteins between wheat and model plant species, allowing us to speculate on putative TaARF functions, informed by the functional clades currently described in *Arabidopsis* (Guilfoyle and Hagen, 2007; Chandler, 2016). For instance, the Clade IIb ARFs TaARF4, TaARF12, and TaARF13 clustered with *Arabidopsis* ARF1/2 (**Figure 1**), suggesting a putative function in flower development and leaf senescence. Likewise, TaARF9, TaARF23, and TaARF24 grouped with *Arabidopsis* ARF13, whose encoding gene is highly expressed in the embryo and the primary root tip. In Clade I, TaARF7, TaARF14, TaARF16, and TaARF22 clustered with *Arabidopsis* ARF6/8 (**Figure 1**), indicative of a role in flower development and the growth of vegetative tissues. The bread wheat ARFs TaARF6 and TaARF18 clustered with *Arabidopsis* ARF5 and ARF19, which regulate embryo and vascular tissue development and lateral root growth. In Clade III, TaARF1, TaARF5, TaARF8, and TaARF21 grouped with *Arabidopsis* ARF10 and ARF17 (**Figure 1**), which are involved in the regulation of root cap cell growth and primexine formation. Although we hypothesize that these orthologous groups represent conserved functions in wheat and *Arabidopsis*, their underlying functions might also have diversified in one species after gene duplication. In this study, the clustering of rice genes in Clades IIb and III indicates that the Triticeae underwent two tandem duplications (*TaARF12* and *TaARF13*, *TaARF23-A.1* and *TaARF23-A.2*) after their evolutionary separation from rice, but before the separation of the three wheat progenitor species. Other segmental duplication events (*TaARF16* and *TaARF22*) clustered with a duplicated block (*OsARF6* and *OsARF17*) in rice (Wang et al., 2007), indicating that the duplication may have occurred before the evolutionary separation from rice (**Figure 1** and **Supplementary Table S3**). From the results mentioned above, it is clear that segmental duplications and tandem duplications have contributed to the expansion of the *ARF* gene family in Triticeae.

We performed a MEME motif analysis on all TaARF proteins and detected 10 highly conserved motifs (**Supplementary Figure S1**). Motif 9 corresponds to a partial CTD domain present only in *TaARF* genes from Clade I, whereas all other motifs were conserved across all subgroups. The CTD is a carboxy-terminal dimerization domain that is essential for auxin responses, indicating that TaARFs that belong to Clade I may be associated with the auxin signaling pathway (Roosjen et al., 2018). Comparing the gene structures and encoded protein domain architecture of *ARF* genes in wheat and rice, the number of introns in rice *ARF* genes varied from 2 to 14, which is consistent with the gene structure of *TaARF* genes (**Figure 2**). These results indicate that each *ARF* gene clade, as defined by the phylogenetic tree, harbors a similar gene structure and domain composition, demonstrating that the protein architecture is extremely conserved within a specific subfamily of *ARF* genes in wheat (**Figure 2C**).

### Functions of *TaARF* Genes in Abiotic Stress and ABA Responses

Phytohormones are essential for the ability of plants to cope with abiotic stress such as salinity, drought, and temperature stresses by mediating a wide range of adaptive responses (Weijers and Wagner, 2016). For example, the plant phytohormone ABA is an important regulator of plant responses to abiotic stress. Previous studies have reported that the mechanisms underlying the transcriptional regulation of dehydration, cold, and salinity stresses include both ABA-dependent and ABA-independent pathways. Within a signal transduction pathway, many transcription factors and stress-responsive *cis*-regulatory elements function as molecular switches for gene expression (Tuteja, 2007). In agreement with this, we observed a modulation in the transcript levels of a subset of *TaARF* genes during stress, as well as the presence of multiple LTRs, DREs, salt-responsive *cis*-elements, and ABREs in *TaARF* promoters.

Numerous *ARF* genes are regulated by miRNAs in response to abiotic stress: for example, miRNA167, which negatively regulates *ARF6* and *ARF8* transcript levels, is induced by drought in *Arabidopsis via* ABA signaling (Liu et al., 2007). Thus, it is worth noting that our results showed that a subset of *TaARF* genes were induced in response to drought stress and ABA treatment. Phylogenetic analysis revealed a sub-clade within Clade I that consisted of four TaARF and two *Arabidopsis* ARF proteins, with TaARF14, TaARF16, and TaARF22 being likely orthologs of *Arabidopsis* ARF6, whereas TaARF7 is a putative ortholog of *Arabidopsis* ARF8. *TaARF14* and *TaARF16* are potential targets of tae-miR167d, and showed a gradual decrease in their transcript levels after an initial induction during drought stress, whereas they were both induced by ABA treatment (**Supplementary Figures S4**, **S5**). Similarly, *TaARF7* was targeted by tae-miR167 and *TaARF22* lacked the tae-miR167d target site, *TaARF22* was induced 11-fold in response to ABA treatment and 60-fold under drought stress, and *TaARF7* transcript levels followed the same trend (**Supplementary Figures S4**, **S5**).

In agreement with their transcriptional responses, the promoter sequences of these *TaARF* genes contained a number of ABREs and DREs (**Figure 4A**), indicating that these *TaARFs* may have potential roles in the crosstalk between drought and ABA signaling pathways (Jaillais and Chory, 2010). In *rabidopsis*, *ARF10* is negatively regulated by miRNA160 and affects ABA sensitivity during seed germination (Liu et al., 2008), and auxin enhances ABA signaling through *ARF10*- and *ARF16*-mediated *ABSCISIC ACID INSENSITIVE* (*ABI3*) activation to control lateral root formation and seed dormancy (Brady et al., 2003; Liu et al., 2013). The wheat ortholog *TaARF1* was predicted to be targeted by tae*-*miR160 and showed a significant down-regulation upon ABA treatment (**Supplementary Figure S5**). This result suggests the conservation of the ARF1–miR160 module between wheat and *Arabidopsis*.

In addition, we identified *TaARF* candidate genes that may be harnessed for enhancing abiotic stress tolerance in plants. During NaCl treatment, *TaARF10* and *TaARF2* displayed 42- to 53-fold higher expression (**Supplementary Figure S3**). *TaARF4* was the gene most highly induced by ABA treatment and cold stress, by 35- and 120-fold, respectively (**Supplementary Figures S5**, **S6**). These results collectively revealed that several *TaARF* genes were induced by multiple abiotic stresses and ABA treatment, suggesting that they may mediate both abiotic stress and ABA responses, and thus further hinting at a role of *TaARFs* in crosstalk between abiotic stress responses and the ABA signaling pathway. Moreover, a majority of *TaARFs* respond to abiotic stress and ABA treatment, indicating that the *TaARF* gene family might have substantial functional redundancy.

Moreover, 70–80% of *TaARF* homoeologous groups (consisting of A, B, and D genome copies) showed non-balanced expression patterns in seedlings when exposed to stresses, with higher or lower expression level from a single homoeolog than from the other two (**Figures 6A–D**). These differences between homoeologs might be associated with epigenetic changes affecting DNA methylation or histone modifications and transposable element insertions in promoters or more varied *cis*-regulatory elements (Edger et al., 2017).

### Tissue-Preferential Expression of *TaARF* Genes

Recent studies have revealed that *ARF* genes exhibit great diversity in their expression patterns between different plants and different organs to exert specific biological functions. The analysis of *ARF* genes in *Arabidopsis* has demonstrated that their expression patterns are independent of their phylogenetic relationships (Okushima et al., 2005; Guilfoyle and Hagen, 2007). This also appears to be the case in wheat, because Clade I orthologs are differentially expressed. Tissue-speciﬁc expression patterns have been observed for *Arabidopsis* Clade I *ARF* genes, and include *ARF6* in developing ﬂowers, *ARF8* in seedlings and flowers, *ARF5* in embryos and vascular tissues and *ARF19* is probably expressed in roots and seedlings. The orthologs of these *ARF* genes in wheat are also differentially expressed: *TaARF14* (*ARF6* ortholog) is preferentially expressed in anthers, *TaARF14* (*ARF8* ortholog) in vegetative tissues and anthers, *TaARF6* (*ARF5* ortholog) in male reproductive tissues and leaves, and *TaARF18* (*ARF19* ortholog) in roots and developing reproductive structures. In some cases, the expression patterns of *ARFs* in wheat and *Arabidopsis* are similar. Among ARFs in Clade IIb, *ARF1* and *ARF2* are highly expressed in developing ﬂoral organs; similarly, *TaARF4* (*ARF1* ortholog), *TaARF12* and *TaARF13* (*ARF2* ortholog) are both preferentially expressed in anthers. Taken together, these results suggest that *TaARF* functions may have diversiﬁed in a similar manner to those of *Arabidopsis ARFs*.

It is worth noting that *TaARF* gene expression increased globally and gradually during anther development (**Figure 5**). We detected a pollen-specific *cis*-element (POLLEN1LELAT52) in the promoters of these genes, consistent with their anther-specific expression pattern. For instance, 11 POLLEN1LELAT52 elements were found in the promoter of *TaARF8-B* alone, which showed single-homoeolog dominance with 1,269-fold higher expression in anthers relative to roots (**Figures 4A**, **5**). Genes encoding phylogenetically related TaARFs share similar expression profiles and may be involved in regulating similar anther developmental processes in wheat. Additionally, some *TaARF* genes exhibited non-flower expression, for example high expression in leaves as well as anthers for *TaARF5*, *TaARF10*, *TaARF17*, and *TaARF19* and ubiquitous expression for *TaARF3*, *TaARF16*, and *TaARF18*.

### Potential Roles of Anther-Preferential *TaARF* Genes in TGMS Wheat

Published work on *ARF* genes has often focused on reproductive development at the expense of other physiological processes. TGMS wheat lines such as BS366 and BS1088 are valuable materials for exploration of the mechanism of male sterility. Previous work revealed disorganization of the phragmoplast and the cell plate, as well as the aberrant separation of dyads during male meiosis I, under cold stress in BS366 (Tang et al., 2011). As previously shown, tae-miR167d/TAS3a-59D6(+) and the associated changes in target *ARF* transcript levels largely controlled conditional male sterility in the TGMS line BS366. Three out of nine *ARF* genes showed differential transcript levels in anthers of the wheat TGMS line BS366 under low temperatures. Contig1892 (identical to *TaARF3* in this study) and Contig4296 (*TaARF11*) are targets of TAS3a-59D6(+), whereas Contig9875 (*TaARF14*) is a target of tae-miR167d (Tang et al., 2011; Tang et al., 2012). Our results showed that *TaARF3*, *TaARF11*, and *TaARF14* transcript levels were induced at the stage 7 under low temperature, which is consistent with previous work (**Supplementary Figure S7**). Moreover, we determined the diverse expression profiles of anther-preferential *TaARF* genes in TGMS lines (**Supplementary Figures S7**, **S8**), suggesting that these genes may play a broad role during male reproduction development. The expression level of *TaARF13* was repressed in TGMS wheat BS366 under low temperature, whereas *TaARF14* was induced. *Arabidopsis ARF2* and *ARF6*, which are reported to participate in flower development and ripening, clustered with the anther-specific wheat genes *TaARF13* and *TaARF14* in Clade IIb and Clade I, respectively, suggesting that *TaARF13* and *TaARF14* are likely required to promote stamen development.

In *Arabidopsis*, ARF17 directly binds to the promoter of *CALLOSE SYNTHASE 5* (*CALS5*), thereby enhancing its expression and regulating callose deposition and pollen viability (Dong et al., 2005). In wheat TMGS lines, the expression level of *TaARF8* (*ARF17* ortholog) dropped significantly in anthers at the stage 8, by at least ~7-fold and 10-fold in the BS366 and BS1088 lines, respectively, at the restrictive temperature. However, *TaARF8* was up-regulated at stage 8 in the cold-treated J411 controls, suggesting that *TaARF8* plays a key role in cold-induced male sterility (**Figure 7B**). *TaCALS5* (*CALS5* ortholog) is highly expressed in anthers at stages 7 and 9 (**Supplementary Figure S9A**). Transcript levels for *TaCALS5* strongly decreased in low temperature with respect to those in the control conditions (**Supplementary Figure S9B**), an expression pattern shared by *TaARF8*. These results suggest that the regulation of wheat *TaARF8*-*TaCALS5* and *Arabidopsis ARF17*-*CALS5* might be conserved and may play a key role in cold-induced male sterility.

By contrast, *TaARF9* showed a divergent spatial-temporal expression pattern from its *Arabidopsis* ortholog *ARF18*, which showed ubiquitous expression. *TaARF9* was preferentially expressed in anthers and was gradually up-regulated from stage 6 to stage 7 under low temperature in both BS366 and BS1088 lines, but showed no signiﬁcant changes in J411 controls (**Figure 7B**), suggesting *TaARF9* as a potential candidate gene for thermosensitive genic male sterility in wheat. *TaARF21* clustered with *Arabidopsis ARF10*, which involved in root cap cell differentiation and ARF10 is known to interact with ARF18 (Li et al., 2011; Szklarczyk et al., 2019). *TaARF21* was induced at stage 8 (**Figure 7B**) under low temperature in TGMS lines, but followed a similar expression pattern in the J411 control, which indicated that *TaARF21* only responds to cold-regulated pathways. In both cases, these observations indicate that *TaARF9* and *TaARF21* may play divergent roles from their orthologs in *Arabidopsis*. Moreover, *TaARF9* was co-expressed with *TaARF21* in TGMS lines, and their encoded proteins shared the same predicted nuclear localization (**Supplementary Table S1**). Thus, we speculate that TaARF9 might interact with TaARF21 to activate the downstream signaling components that regulate the cold-induced male sterility pathway.

## Conclusion

The present study provides a comprehensive analysis of the 69 wheat *ARF* genes, grouped in 24 homoeologous groups. Phylogenetic analysis and structural characteristics revealed the evolutionary conservation and variation of *ARF* genes among different plant species. Chromosomal distribution and duplications of the *TaARF* family members provided valuable information about the evolutionary aspects of the wheat genome. A comprehensive analysis of *TaARF* gene expression patterns give support for various functional roles of ARFs in abiotic stress and developmental processes. These analyses also provide an overview of the relative homoeolog expression level bias during stresses and plant development. Additionally, the identiﬁcation of anther-preferential expression of *TaARF8*, *TaARF9*, and *TaARF21* in response to cold stress in TGMS lines suggests that these candidate genes as potentially regulate male sterility. Taken together, our research provides significant insight for further investigating the role of members of the wheat *ARF* gene family in abiotic stress signaling and male reproductive development.

## Data Availability Statement

All datasets presented in this study are included in the article/**Supplementary Material**.

## Author Contributions

YT and CZ designed the study. LX and DW performed the experiments and analyzed the data. SL, ZF, CG, and SS treated and collected experimental materials. LX, YT, and CZ drafted the manuscript. All authors contributed to the article and approved the submitted version.

## Funding

This study was supported by Innovative special reserve project of Beijing Academy of agriculture and Forestry Sciences (KJCX20200423) and Collaborative innovation center of genomics breeding (KJCX201907-2), Beijing Academy of agriculture and Forestry Sciences.

## Conflict of Interest

The authors declare that the research was conducted in the absence of any commercial or financial relationships that could be construed as a potential conflict of interest.

## Supplementary Material

Supplementary Figure 1 Conserved motif analysis of the wheat ARF protein family, identified through MEME suite.

Supplementary Figure 2 *Cis*-element identification and distribution in the promoters of *TaARFs*. Colored regions show classifications of *TaARF* genes in the neighbor-joining tree.

Supplementary Figure 3 Expression patterns of *TaARF* genes under salt stress. We treated 14-day-old wheat seedlings with 250 mM NaCl for 0, 1, 2, 5, 10, and 24 h. Relative expression of each *TaARF* gene was normalized to *TaACTIN*. Data represent the mean of three biological replicates; error bars represent the standard error.

Supplementary Figure 4 Expression patterns of *TaARF* genes under drought stress. We treated 14-day-old wheat seedlings with PEG-6000 (25%, w/v) for 0, 1, 2, 5, 10, and 24 h. Relative expression of each *TaARF* gene was normalized to *TaACTIN*. Data represent the mean of three biological repeats; error bars represent the standard error.

Supplementary Figure 5 Expression patterns of *TaARF* genes under ABA treatment. We treated 14-day-old wheat seedlings with 200 µM ABA for 0, 1, 2, 5, 10, and 24 h. Relative expression of each *TaARF* gene was normalized to *TaACTIN*. Data represent the mean of three biological repeats; error bars represent the standard error.

Supplementary Figure 6 Expression patterns of *TaARF* genes during exposure to cold stress. We exposed 14-day-old wheat seedlings to 4°C for 0, 1, 2, 5, 10, and 24 h. Relative expression of each *TaARF* gene was normalized to *TaACTIN*. Data represent the mean of three biological repeats, error bars represent the standard error.

Supplementary Figure 7 Expression profiles of *TaARF* genes in the anthers of TGMS line BS366 under control conditions (control) and low temperature treatment (LT). S6: stage 6, central callose stage; S7: stage 7, meiotic stage; S8: stage 8, tetrad stage; S9: stage 9, young microspore stage. Data represent the mean of three biological repeats; error bars represent the standard error.

Supplementary Figure 8 Expression profiles of anther-specific *TaARF* genes in the anthers of TGMS line BS1088 under control condition (control) and low temperature treatment (LT). S6: stage 6, central callose stage; S7: stage 7, meiotic stage; S8: stage 8, tetrad stage; S9: stage 9, young microspore stage. Data represent the mean of three biological repeats; error bars represent the standard error.

Supplementary Figure 9 Expression patterns of *TaCalS5*. **(A)** *TaCalS5* is specifically expressed in anthers. **(B)** Relative transcript levels of *TaCalS5* in the anthers of TGMS wheat lines under control conditions (control) and low-temperature treatment (LT). S6: stage 6, central callose stage; S7: stage 7, meiotic stage; S8: stage 8, tetrad stage; S9: stage 9, young microspore stage. Data represent the mean of three biological repeats; error bars represent the standard error.

## Abbreviations

ARF, auxin response factor; ABA, abscisic acid; RT-qPCR, real-time quantitative PCR; TGMS, thermosensitive genic male sterile; LT, low temperature.

## References

[B1] Attia K. A. Abdelkhalik A. F. Ammar M. H. Wei C. Yang J. Lightfoot D. A. (2009). Antisense phenotypes reveal a functional expression of OsARF1, an auxin response factor, in transgenic rice. Curr. Issues Mol. Biol. 11 (Suppl 1), i29–i34. 10.21775/9781912530069.04 19193962

[B2] Brady S. M. Sarkar S. F. Bonetta D. McCourt P. (2003). The ABSCISIC ACID INSENSITIVE 3 (ABI3) gene is modulated by farnesylation and is involved in auxin signaling and lateral root development in Arabidopsis. Plant J. 34, 67–75. 10.1046/j.1365-313x.2003.01707.x 12662310

[B3] Browne R. G. Iacuone S. Li S. F. Dolferus R. Parish R. W. (2018). Anther Morphological Development and Stage Determination in Triticum aestivum. Front. Plant Sci. 9, 288. 10.3389/fpls.2018.00228 29527219 PMC5829449

[B4] Burks D. J. Azad R. K. (2016). Identification and Network-Enabled Characterization of Auxin Response Factor Genes in Medicago truncatula. Front. Plant Sci. 7, 1857. 10.3389/fpls.2016.01857 28018393 PMC5145899

[B5] Chandler J. W. (2016). Auxin response factors: Auxin response factors. Plant Cell Environ. 39, 1014–1028. 10.1111/pce.12662 26487015

[B6] Dong X. Hong Z. Sivaramakrishnan M. Mahfouz M. Verma D. P. S. (2005). Callose synthase (CalS5) is required for exine formation during microgametogenesis and for pollen viability in Arabidopsis. Plant J. Cell Mol. Biol. 42, 315–328. 10.1111/j.1365-313X.2005.02379.x 15842618

[B7] D’Ario M. Griffiths-Jones S. Kim M. (2017). Small RNAs: Big Impact on Plant Development. Trends Plant Sci. 22, 1056–1068. 10.1016/j.tplants.2017.09.009 29032035

[B8] Edger P. P. Smith R. McKain M. R. Cooley A. M. Vallejo-Marin M. Yuan Y. (2017). Subgenome Dominance in an Interspecific Hybrid, Synthetic Allopolyploid, and a 140-Year-Old Naturally Established Neo-Allopolyploid Monkeyflower. Plant Cell 29, 2150–2167. 10.1105/tpc.17.00010 28814644 PMC5635986

[B9] Finet C. Berne-Dedieu A. Scutt C. P. Marletaz F. (2013). Evolution of the ARF Gene Family in Land Plants: Old Domains, New Tricks. Mol. Biol. Evol. 30, 45–56. 10.1093/molbev/mss220 22977118

[B10] Ghelli R. Brunetti P. Napoli N. Paolis A. D. Cecchetti V. Tsuge T. (2018). A Newly Identified Flower-Specific Splice Variant of AUXIN RESPONSE FACTOR8 Regulates Stamen Elongation and Endothecium Lignification in Arabidopsis. Plant Cell 30, 620–637. 10.1105/tpc.17.00840 29514943 PMC5894849

[B11] Gill B. S. Appels R. Botha-Oberholster A.-M. Buell C. R. Bennetzen J. L. Chalhoub B. (2004). A workshop report on wheat genome sequencing: International Genome Research on Wheat Consortium. Genetics 168, 1087–1096. 10.1534/genetics.104.034769 15514080 PMC1448818

[B12] Guilfoyle T. J. Hagen G. (2007). Auxin response factors. Curr. Opin. Plant Biol. 10, 453–460. 10.1016/j.pbi.2007.08.014 17900969

[B13] Gutierrez L. Bussell J. D. Păcurar D. II Schwambach J. Păcurar M. Bellini C. (2009). Phenotypic Plasticity of Adventitious Rooting in Arabidopsis Is Controlled by Complex Regulation of AUXIN RESPONSE FACTOR Transcripts and MicroRNA Abundance. Plant Cell 21, 3119–3132. 10.1105/tpc.108.064758 19820192 PMC2782293

[B14] Ha C. V. Le D. T. Nishiyama R. Watanabe Y. Sulieman S. Tran U. T. (2013). The auxin response factor transcription factor family in soybean: genome-wide identification and expression analyses during development and water stress. DNA Res. Int. J. Rapid Publ. Rep. Genes Genomes 20, 511–524. 10.1093/dnares/dst027 PMC3789561 23810914

[B15] Hannah M. A. Heyer A. G. Hincha D. K. (2005). A global survey of gene regulation during cold acclimation in Arabidopsis thaliana. PloS Genet. 1, e26. 10.1371/journal.pgen.0010026 16121258 PMC1189076

[B16] Hu W. Zuo J. Hou X. Yan Y. Wei Y. Liu J. (2015). The auxin response factor gene family in banana: genome-wide identification and expression analyses during development, ripening, and abiotic stress. Front. Plant Sci. 6, 742. 10.3389/fpls.2015.00742 26442055 PMC4569978

[B17] Jaillais Y. Chory J. (2010). Unraveling the paradoxes of plant hormone signaling integration. Nat. Struct. Mol. Biol. 17, 642–645. 10.1038/nsmb0610-642 20520656 PMC3166629

[B18] Krogan N. T. Berleth T. (2012). A dominant mutation reveals asymmetry in MP/ARF5 function along the adaxial-abaxial axis of shoot lateral organs. Plant Signal. Behav. 7, 940–943. 10.4161/psb.20790 22751359 PMC3474690

[B19] Krogan N. T. Yin X. Ckurshumova W. Berleth T. (2014). Distinct subclades of Aux/IAA genes are direct targets of ARF5/MP transcriptional regulation. New Phytol. 204, 474–483. 10.1111/nph.12994 25145395

[B20] Li J.-F. Bush J. Xiong Y. Li L. McCormack M. (2011). Large-scale protein-protein interaction analysis in Arabidopsis mesophyll protoplasts by split firefly luciferase complementation. PloS One 6, e27364. 10.1371/journal.pone.0027364 22096563 PMC3212559

[B21] Li S.-B. OuYang W.-Z. Hou X.-J. Xie L.-L. Hu C.-G. Zhang J.-Z. (2015). Genome-wide identification, isolation and expression analysis of auxin response factor (ARF) gene family in sweet orange (Citrus sinensis). Front. Plant Sci. 6:119. 10.3389/fpls.2015.00119 25870601 PMC4378189

[B22] Li S.-B. Xie Z.-Z. Hu C.-G. Zhang J.-Z. (2016). A Review of Auxin Response Factors (ARFs) in Plants. Front. Plant Sci. 7, 47. 10.3389/fpls.2016.00047 26870066 PMC4737911

[B23] Liu P.-P. Montgomery T. A. Fahlgren N. Kasschau K. D. Nonogaki H. Carrington J. C. (2007). Repression of AUXIN RESPONSE FACTOR10 by microRNA160 is critical for seed germination and post-germination stages. Plant J. Cell Mol. Biol. 52, 133–146. 10.1111/j.1365-313X.2007.03218.x 17672844

[B24] Liu H.-H. Tian X. Li Y.-J. Wu C.-A. Zheng C.-C. (2008). Microarray-based analysis of stress-regulated microRNAs in Arabidopsis thaliana. RNA N. Y. N. 14, 836–843. 10.1261/rna.895308 PMC2327369 18356539

[B25] Liu X. Zhang H. Zhao Y. Feng Z. Li Q. Yang H.-Q. (2013). Auxin controls seed dormancy through stimulation of abscisic acid signaling by inducing ARF-mediated ABI3 activation in Arabidopsis. Proc. Natl. Acad. Sci. U.S.A. 110, 15485–15490. 10.1073/pnas.1304651110 23986496 PMC3780901

[B26] Marin E. Jouannet V. Herz A. Lokerse A. S. Weijers D. Vaucheret H. (2010). miR390, Arabidopsis TAS3 tasiRNAs, and Their AUXIN RESPONSE FACTOR Targets Define an Autoregulatory Network Quantitatively Regulating Lateral Root Growth. Plant Cell 22, 1104–1117. 10.1105/tpc.109.072553 20363771 PMC2879756

[B27] Nagpal P. Ellis C. M. Weber H. Ploense S. E. Barkawi L. S. Guilfoyle T. J. (2005). Auxin response factors ARF6 and ARF8 promote jasmonic acid production and flower maturation. Dev. Camb. Engl. 132, 4107–4118. 10.1242/dev.01955 16107481

[B28] Okushima Y. Overvoorde P. J. Arima K. Alonso J. M. Chan A. Chang C. (2005). Functional Genomic Analysis of the AUXIN RESPONSE FACTOR Gene Family Members in Arabidopsis thaliana: Unique and Overlapping Functions of ARF7 and ARF19. Plant Cell Online 17, 444–463. 10.1105/tpc.104.028316 PMC548818 15659631

[B29] Qi Y. Wang S. Shen C. Zhang S. Chen Y. Xu Y. (2012). OsARF12, a transcription activator on auxin response gene, regulates root elongation and affects iron accumulation in rice (Oryza sativa). New Phytol. 193, 109–120. 10.1111/j.1469-8137.2011.03910.x 21973088

[B30] Qiao L. Zhang W. Li X. Zhang L. Zhang X. Li X. (2018). Characterization and Expression Patterns of Auxin Response Factors in Wheat. Front. Plant Sci. 9:1395. 10.3389/fpls.2018.01395 30283490 PMC6157421

[B31] Ramírez-González R. H. Borrill P. Lang D. Harrington S. A. Brinton J. Venturini L. (2018). The transcriptional landscape of polyploid wheat. Science 361, eaar6089. 10.1126/science.aar6089 30115782

[B32] Roosjen M. Paque S. Weijers D. (2018). Auxin Response Factors: output control in auxin biology. J. Exp. Bot. 69, 179–188. 10.1093/jxb/erx237 28992135

[B33] Shen C. Wang S. Zhang S. Xu Y. Qian Q. Qi Y. (2013). OsARF16, a transcription factor, is required for auxin and phosphate starvation response in rice (Oryza sativa L.). Plant Cell Environ. 36, 607–620. 10.1111/pce.12001 22913536

[B34] Shen C. Yue R. Sun T. Zhang L. Xu L. Tie S. (2015). Genome-wide identification and expression analysis of auxin response factor gene family in Medicago truncatula. Front. Plant Sci. 6:73. 10.3389/fpls.2015.00073 25759704 PMC4338661

[B35] Shi Z.-H. Zhang C. Xu X.-F. Zhu J. Zhou Q. Ma L.-J. (2015). Overexpression of AtTTP Affects ARF17 Expression and Leads to Male Sterility in Arabidopsis. PloS One 10, e0117317. 10.1371/journal.pone.0117317 25822980 PMC4378849

[B36] Spealman P. Naik A. W. May G. E. Kuersten S. Freeberg L. Murphy R. F. (2018). Conserved non-AUG uORFs revealed by a novel regression analysis of ribosome profiling data. Genome Res. 28, 214–222. 10.1101/gr.221507.117 29254944 PMC5793785

[B37] Szklarczyk D. Gable A. L. Lyon D. Junge A. Wyder S. Huerta-Cepas J. (2019). STRING v11: protein-protein association networks with increased coverage, supporting functional discovery in genome-wide experimental datasets. Nucleic Acids Res. 47, D607–D613. 10.1093/nar/gky1131 30476243 PMC6323986

[B38] Tang Z. Zhang L. Yang D. Zhao C. Zheng Y. (2011). Cold stress contributes to aberrant cytokinesis during male meiosis I in a wheat thermosensitive genic male sterile line: Cold stress contributes to aberrant cytokinesis. Plant Cell Environ. 34, 389–405. 10.1111/j.1365-3040.2010.02250.x 21062315

[B39] Tang Z. Zhang L. Xu C. Yuan S. Zhang F. Zheng Y. (2012). Uncovering Small RNA-Mediated Responses to Cold Stress in a Wheat Thermosensitive Genic Male-Sterile Line by Deep Sequencing. Plant Physiol. 159, 721–738. 10.1104/pp.112.196048 22508932 PMC3375937

[B40] The International Wheat Genome Sequencing Consortium (IWGSC) Mayer K. F. X. Rogers J. Dole el J. Pozniak C. Eversole K. (2014). A chromosome-based draft sequence of the hexaploid bread wheat (Triticum aestivum) genome. Science 345, 1251788–1251788. 10.1126/science.1251788 25035500

[B41] Tiwari S. B. Hagen G. Guilfoyle T. (2003). The roles of auxin response factor domains in auxin-responsive transcription. Plant Cell 15, 533–543. 10.1105/tpc.008417 12566590 PMC141219

[B42] Tuteja N. (2007). Abscisic Acid and Abiotic Stress Signaling. Plant Signal. Behav. 2, 135–138. 10.4161/psb.2.3.4156 19516981 PMC2634038

[B43] Ulmasov T. Hagen G. Guilfoyle T. J. (1999a). Activation and repression of transcription by auxin-response factors. Proc. Natl. Acad. Sci. U. S. A. 96, 5844–5849. 10.1073/pnas.96.10.5844 10318972 PMC21948

[B44] Ulmasov T. Hagen G. Guilfoyle T. J. (1999b). Dimerization and DNA binding of auxin response factors. Plant J. Cell Mol. Biol. 19, 309–319. 10.1046/j.1365-313X.1999.00538.x 10476078

[B45] Wang S. Tiwari S. B. Hagen G. Guilfoyle T. J. (2005). AUXIN RESPONSE FACTOR7 restores the expression of auxin-responsive genes in mutant Arabidopsis leaf mesophyll protoplasts. Plant Cell 17, 1979–1993. 10.1105/tpc.105.031096 15923351 PMC1167546

[B46] Wang D. Pei K. Fu Y. Sun Z. Li S. Liu H. (2007). Genome-wide analysis of the auxin response factors (ARF) gene family in rice (Oryza sativa). Gene 394, 13–24. 10.1016/j.gene.2007.01.006 17408882

[B47] Wang M. Yue H. Feng K. Deng P. Song W. Nie X. (2016). Genome-wide identification, phylogeny and expressional profiles of mitogen activated protein kinase kinase kinase (MAPKKK) gene family in bread wheat (Triticum aestivum L.). BMC Genomics 17, 668. 10.1186/s12864-016-2993-7 27549916 PMC4994377

[B48] Weijers D. Wagner D. (2016). Transcriptional Responses to the Auxin Hormone. Annu. Rev. Plant Biol. 67, 539–574. 10.1146/annurev-arplant-043015-112122 26905654

[B49] Wilmoth J. C. Wang S. Tiwari S. B. Joshi A. D. Hagen G. Guilfoyle T. J. (2005). NPH4/ARF7 and ARF19 promote leaf expansion and auxin-induced lateral root formation. Plant J. Cell Mol. Biol. 43, 118–130. 10.1111/j.1365-313X.2005.02432.x 15960621

[B50] Wu M.-F. Tian Q. Reed J. W. (2006). Arabidopsis microRNA167 controls patterns of ARF6 and ARF8 expression, and regulates both female and male reproduction. Development 133, 4211–4218. 10.1242/dev.02602 17021043

[B51] Xu L. Tang Y. Gao S. Su S. Hong L. Wang W. (2016). Comprehensive analyses of the annexin gene family in wheat. BMC Genomics 17, 415. 10.1186/s12864-016-2750-y 27236332 PMC4884362

[B52] Yang J. Tian L. Sun M.-X. Huang X.-Y. Zhu J. Guan Y.-F. (2013). AUXIN RESPONSE FACTOR17 Is Essential for Pollen Wall Pattern Formation in Arabidopsis. Plant Physiol. 162, 720–731. 10.1104/pp.113.214940 23580594 PMC3668065

[B53] Zhang S. Wang S. Xu Y. Yu C. Shen C. Qian Q. (2015). The auxin response factor, OsARF19, controls rice leaf angles through positively regulating OsGH3-5 and OsBRI1. Plant Cell Environ. 38, 638–654. 10.1111/pce.12397 24995795

[B54] Zouine M. Fu Y. Chateigner-Boutin A.-L. Mila I. Frasse P. Wang H. (2014). Characterization of the tomato ARF gene family uncovers a multi-levels post-transcriptional regulation including alternative splicing. PloS One 9, e84203. 10.1371/journal.pone.0084203 24427281 PMC3888382

