# Supplementary figures and images for: Comprehensive Atlas of Wheat (Triticum aestivum L.) AUXIN RESPONSE FACTOR Expression During Male Reproductive Development and Abiotic Stress
Source: Front Plant Sci. 2020 Sep 30;11:586144. doi: 10.3389/fpls.2020.586144 (PMC7554351; doi:10.3389/fpls.2020.586144)

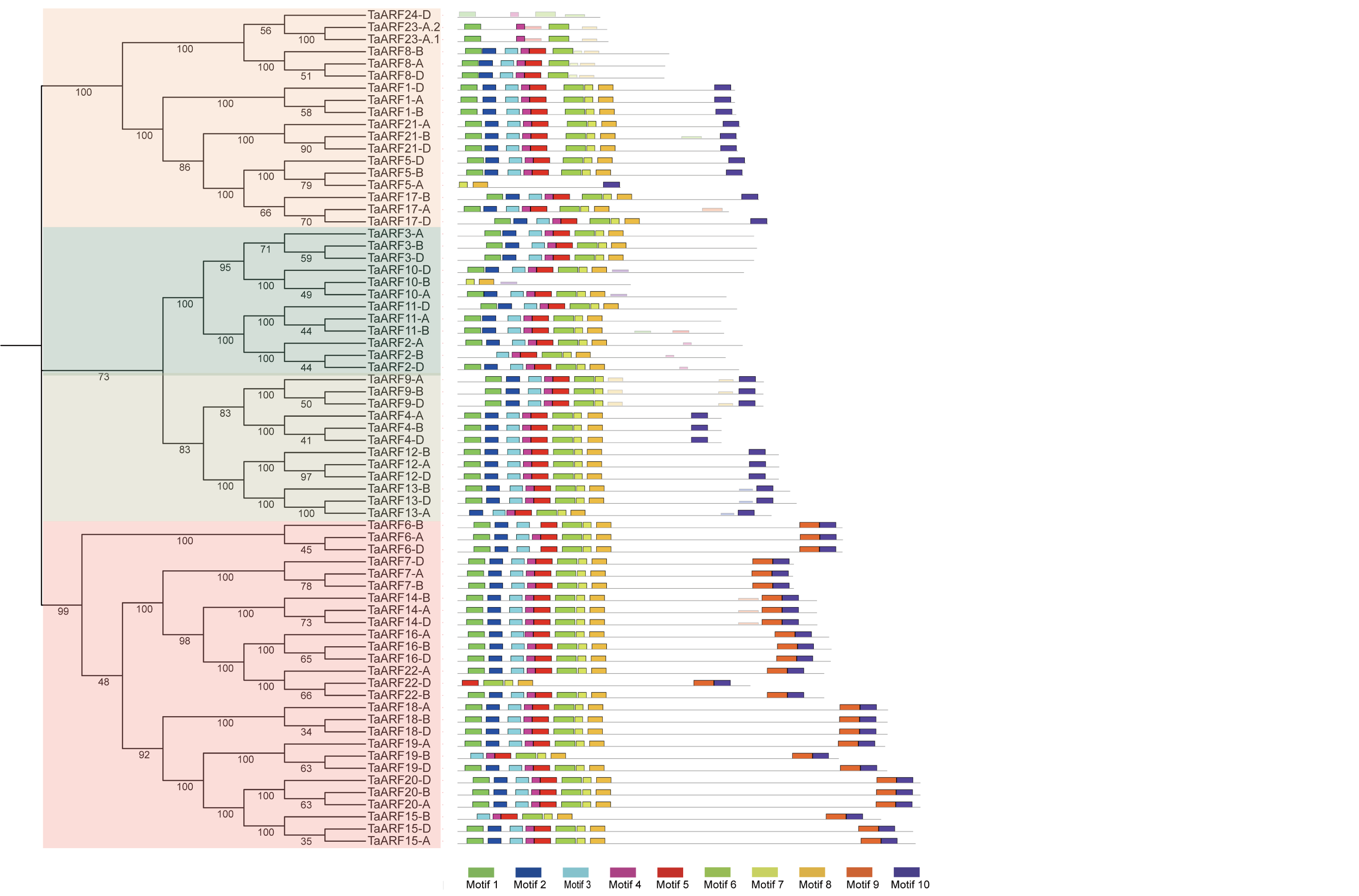

Supplement: Supplementary Figure 1 — Conserved motif analysis of the wheat ARF protein family, identified through MEME suite. [file Image_1.tif]

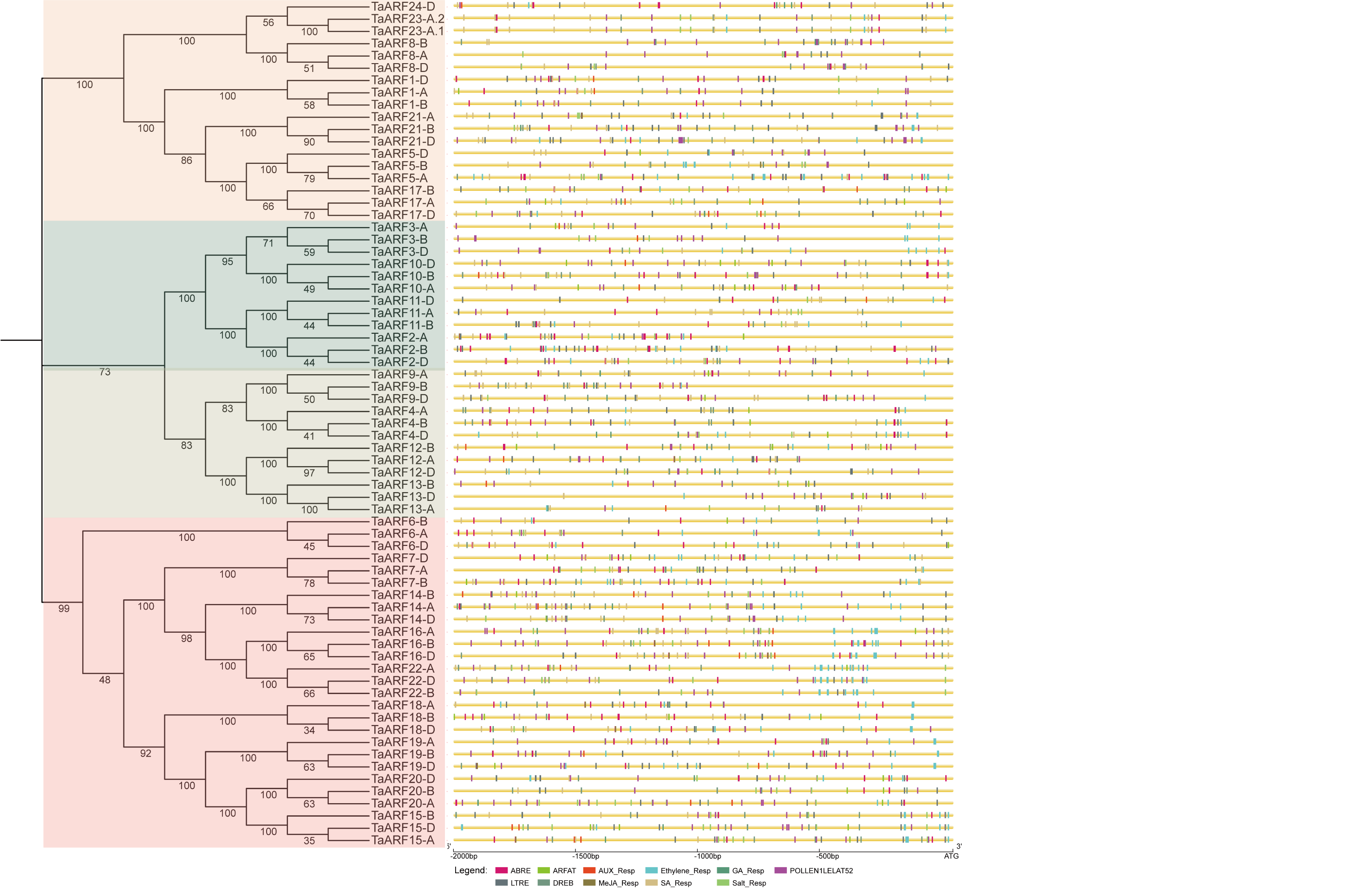

Supplement: Supplementary Figure 2 — Cis-element identification and distribution in the promoters of TaARFs. Colored regions show classifications of TaARF genes in the neighbor-joining tree. [file Image_2.tif]

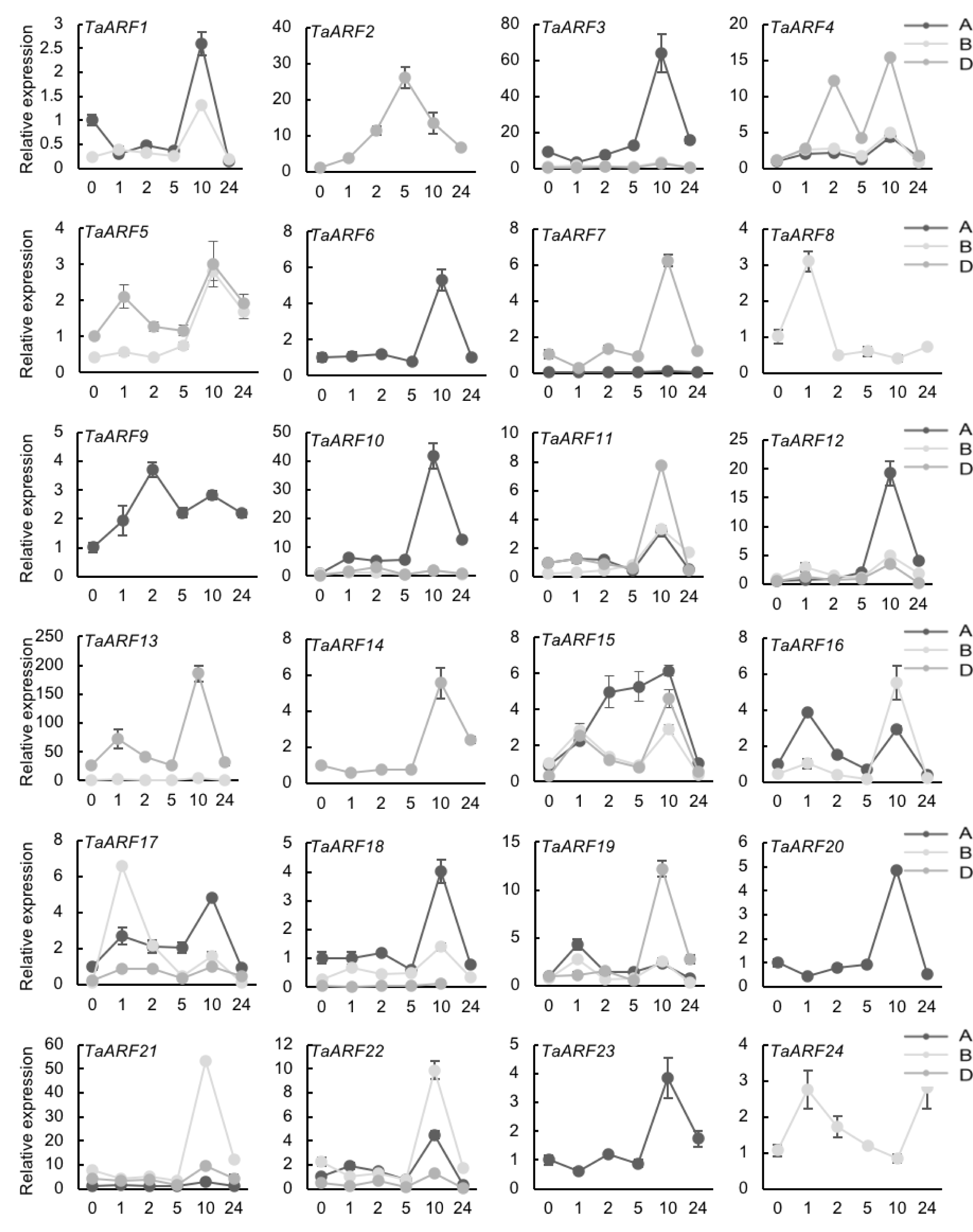

Supplement: Supplementary Figure 3 — Expression patterns of TaARF genes under salt stress. We treated 14-day-old wheat seedlings with 250 mM NaCl for 0, 1, 2, 5, 10, and 24 h. Relative expression of each TaARF gene was normalized to TaACTIN. Data represent the mean of three biological replicates; error bars represent the standard error. [file Image_3.tif]

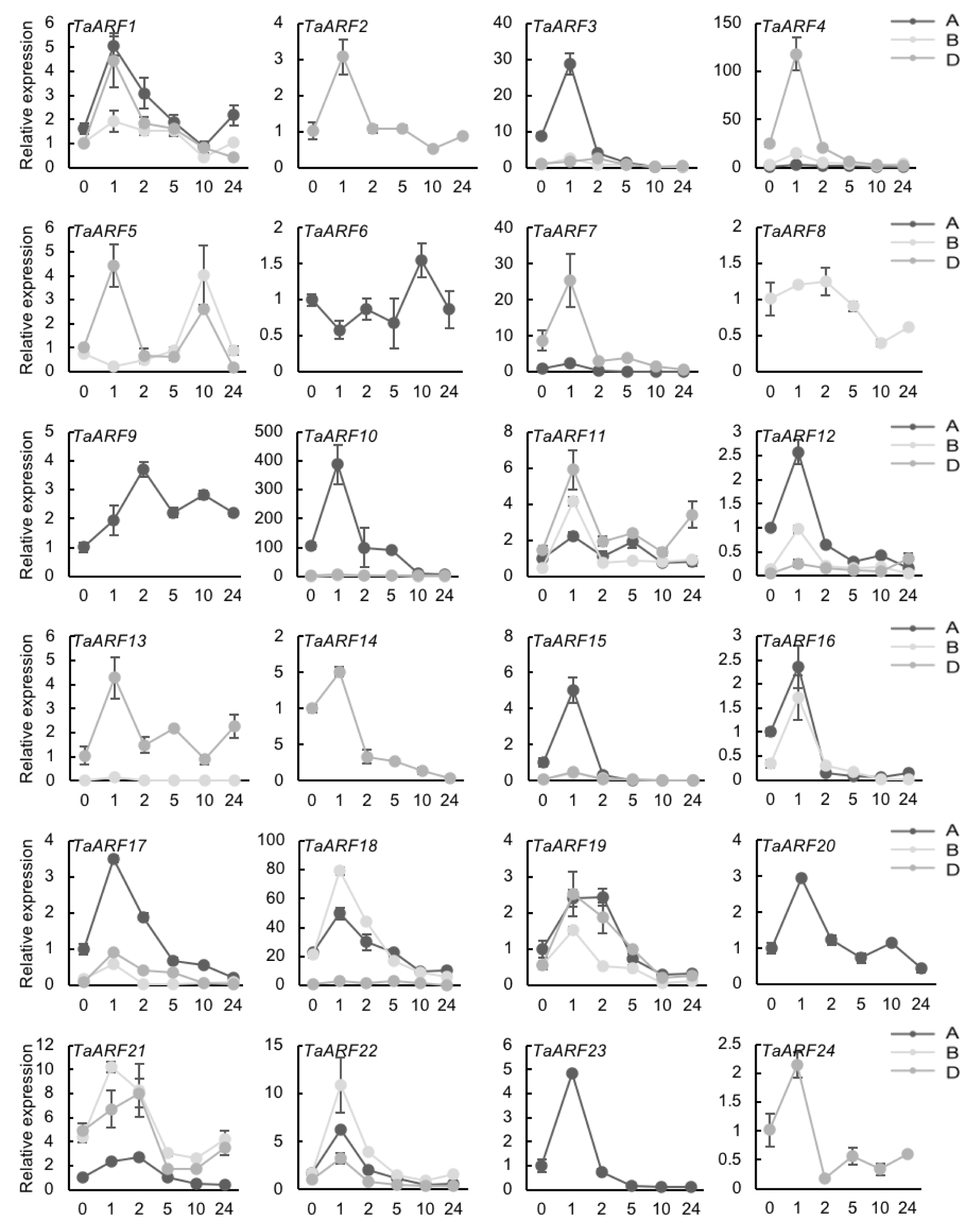

Supplement: Supplementary Figure 4 — Expression patterns of TaARF genes under drought stress. We treated 14-day-old wheat seedlings with PEG-6000 (25%, w/v) for 0, 1, 2, 5, 10, and 24 h. Relative expression of each TaARF gene was normalized to TaACTIN. Data represent the mean of three biological repeats; error bars represent the standard error. [file Image_4.tif]

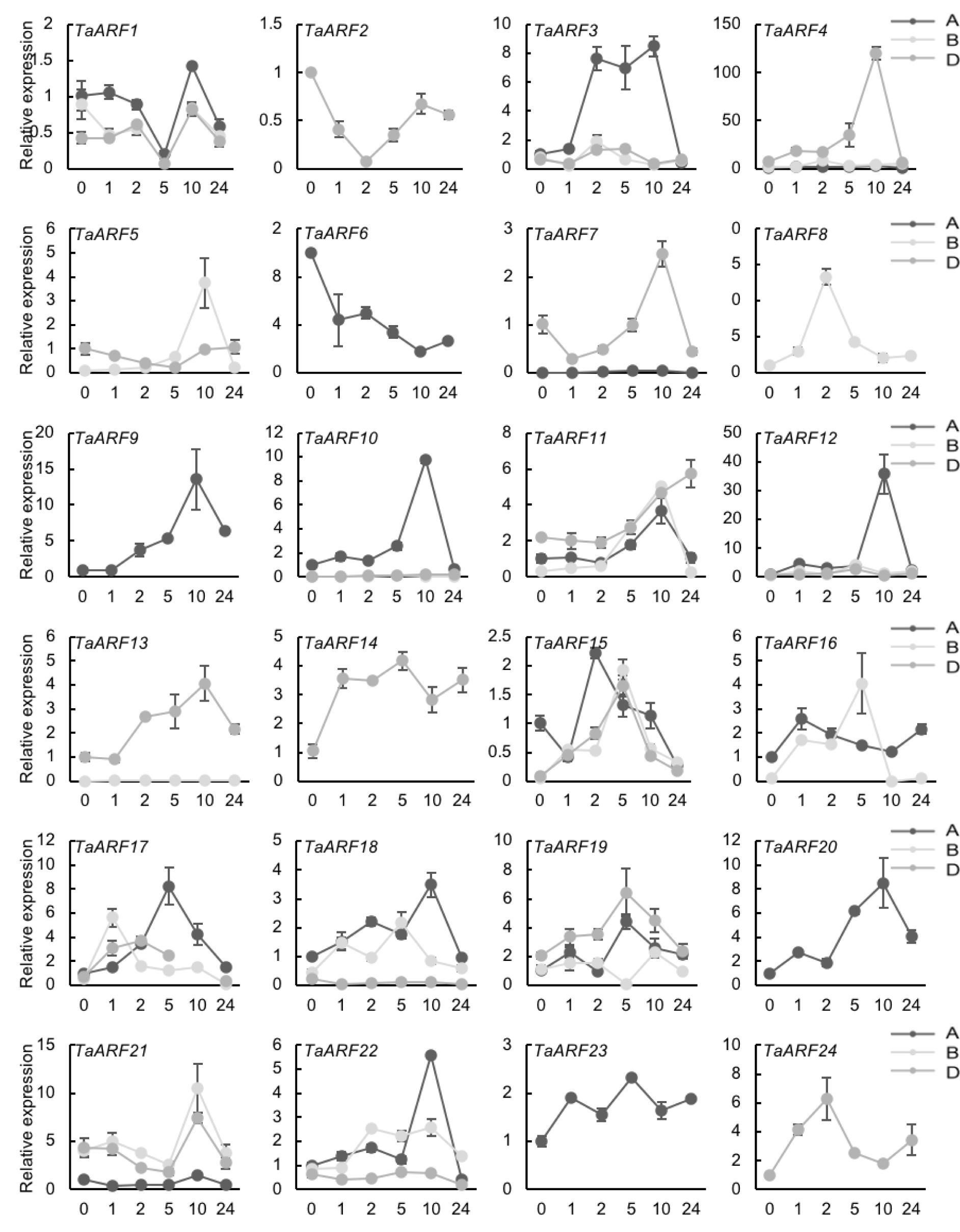

Supplement: Supplementary Figure 5 — Expression patterns of TaARF genes under ABA treatment. We treated 14-day-old wheat seedlings with 200 µM ABA for 0, 1, 2, 5, 10, and 24 h. Relative expression of each TaARF gene was normalized to TaACTIN. Data represent the mean of three biological repeats; error bars represent the standard error. [file Image_5.tif]

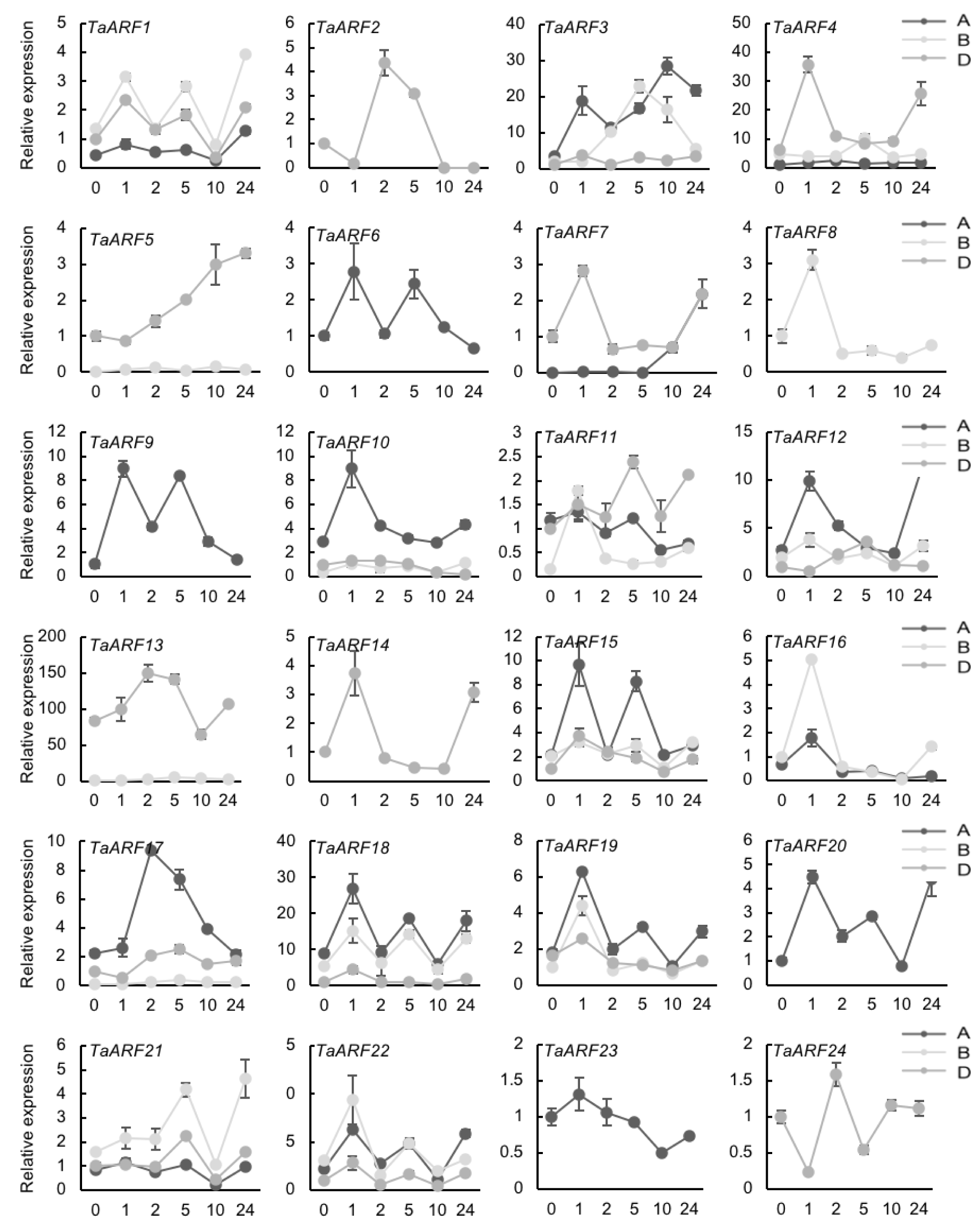

Supplement: Supplementary Figure 6 — Expression patterns of TaARF genes during exposure to cold stress. We exposed 14-day-old wheat seedlings to 4°C for 0, 1, 2, 5, 10, and 24 h. Relative expression of each TaARF gene was normalized to TaACTIN. Data represent the mean of three biological repeats, error bars represent the standard error. [file Image_6.tif]

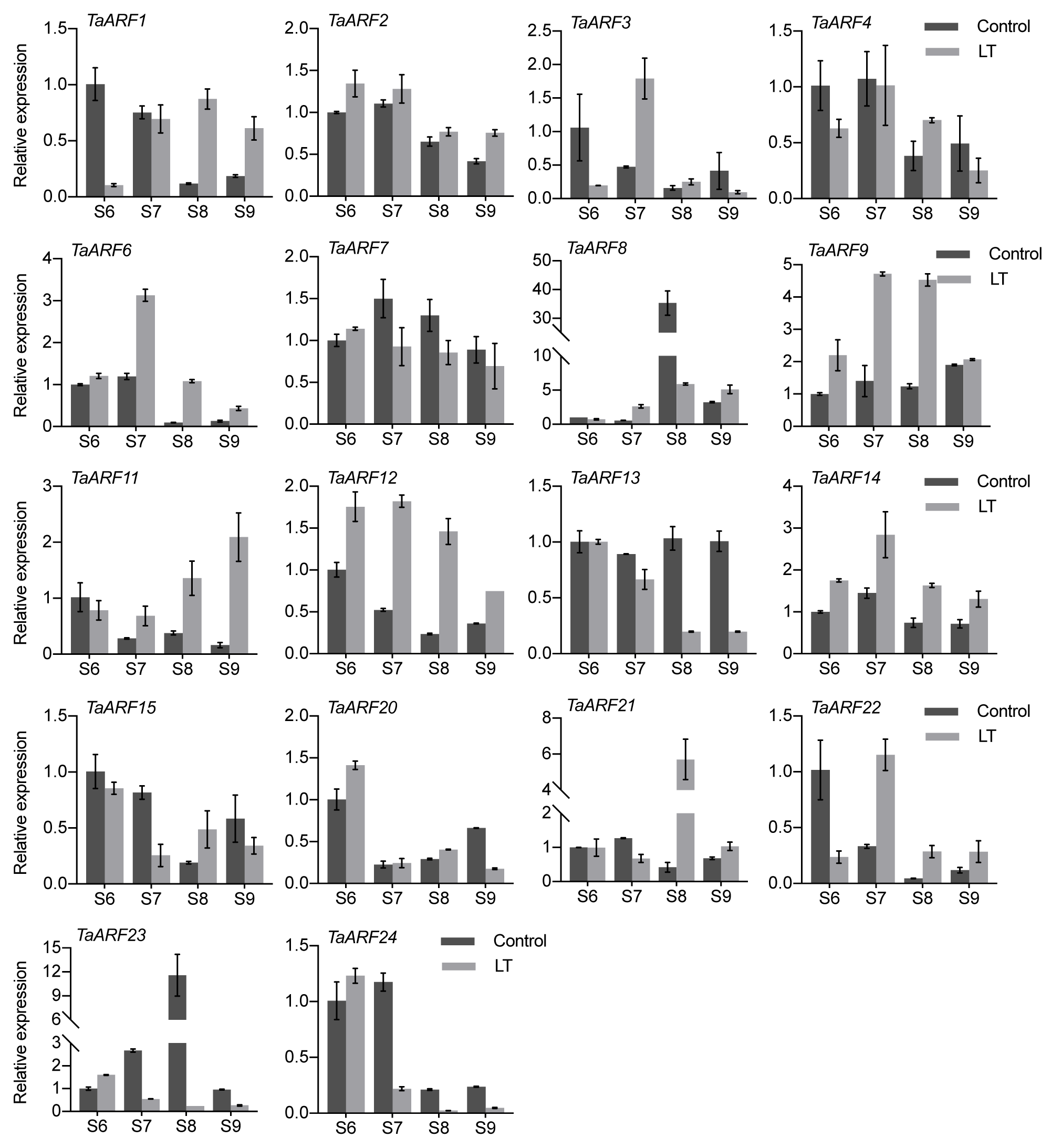

Supplement: Supplementary Figure 7 — Expression profiles of TaARF genes in the anthers of TGMS line BS366 under control conditions (control) and low temperature treatment (LT). S6: stage 6, central callose stage; S7: stage 7, meiotic stage; S8: stage 8, tetrad stage; S9: stage 9, young microspore stage. Data represent the mean of three biological repeats; error bars represent the standard error. [file Image_7.tif]

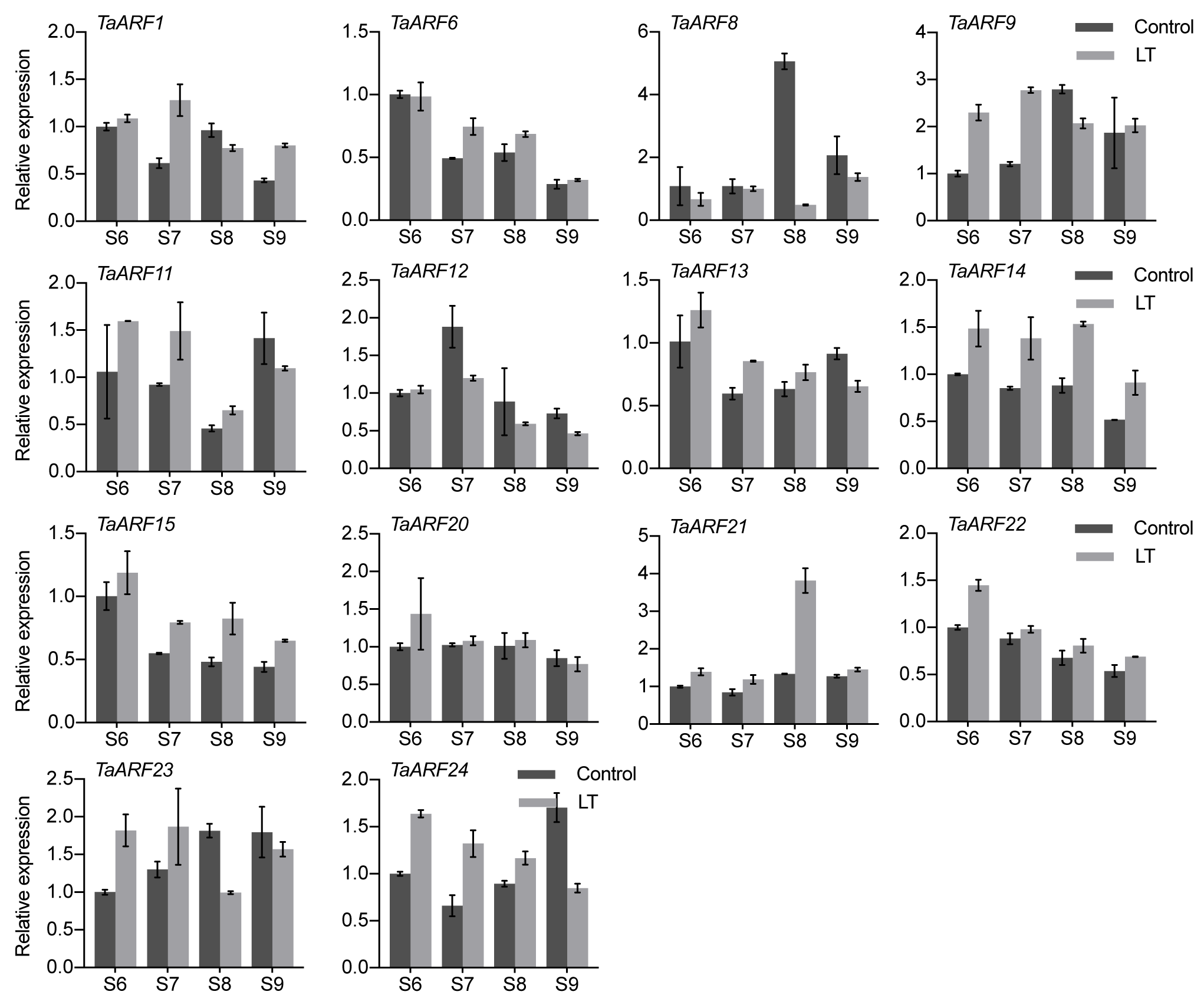

Supplement: Supplementary Figure 8 — Expression profiles of anther-specific TaARF genes in the anthers of TGMS line BS1088 under control condition (control) and low temperature treatment (LT). S6: stage 6, central callose stage; S7: stage 7, meiotic stage; S8: stage 8, tetrad stage; S9: stage 9, young microspore stage. Data represent the mean of three biological repeats; error bars represent the standard error. [file Image_8.tif]

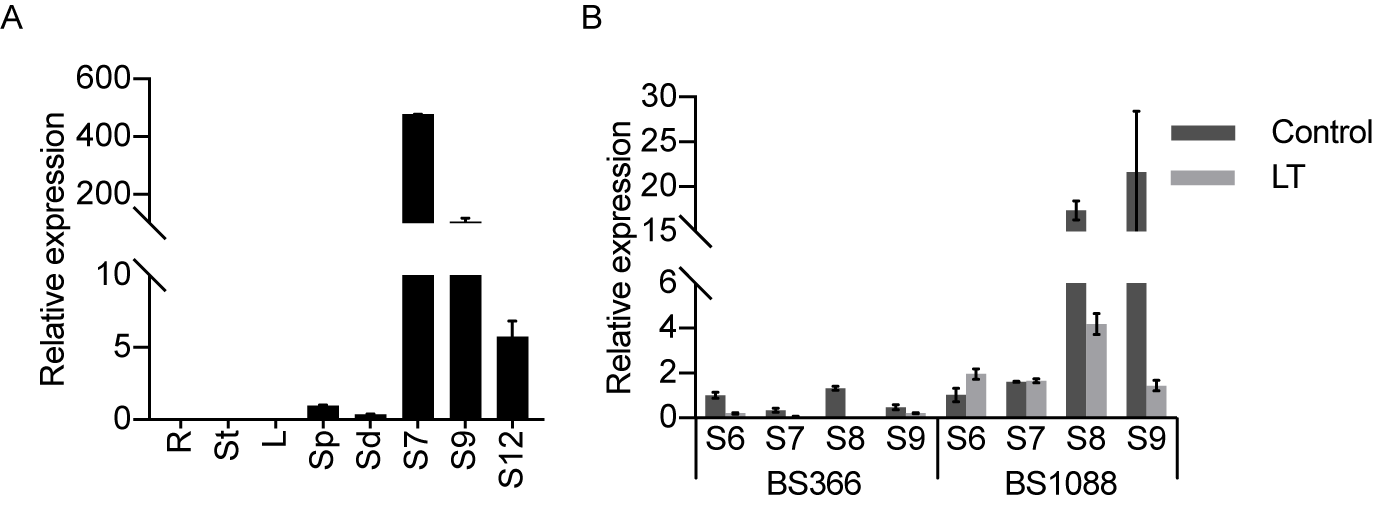

Supplement: Supplementary Figure 9 — Expression patterns of TaCalS5. (A) TaCalS5 is specifically expressed in anthers. (B) Relative transcript levels of TaCalS5 in the anthers of TGMS wheat lines under control conditions (control) and low-temperature treatment (LT). S6: stage 6, central callose stage; S7: stage 7, meiotic stage; S8: stage 8, tetrad stage; S9: stage 9, young microspore stage. Data represent the mean of three biological repeats; error bars represent the standard error. [file Image_9.tif]
